# Physiological Responses and Time-Course Transcriptomic Analysis of *Morchella sextelata* Strain Mel-21 During NaCl Exposure

**DOI:** 10.3390/jof12100736

**Published:** 2026-10-01

**Authors:** Sheng Qiang, Shu-De Yang, Zu-Can Huang, Qi-Meng Zhao, Jing Sun, Tong Yang, Xue-Qi Zhao, Ze-Yang Li, Jun-Yu Zhang, Yu Liu, Jian-Rui Wang

**Affiliations:** College of Horticulture, Ludong University, Yantai 264025, China

**Keywords:** *Morchella*, salt stress, physiological and biochemical responses, WGCNA, *Gene05935*

## Abstract

Salt stress limits fungal growth and distribution, but the mechanisms underlying salt tolerance in *Morchella* remain unclear. Here, we combined physiological measurements, transcriptomic analysis, weighted gene co-expression network analysis (WGCNA), and heterologous expression assays to characterize salt-stress responses in *Morchella sextelata*. Among 24 preselected isolates representing four *Morchella* species, *M. sextelata* Mel-21 obtained the highest composite screening score under 1 mol·L^−1^ NaCl and was selected for detailed analysis. A concentration of 300 mmol·L^−1^ NaCl was subsequently selected as the representative stress condition. Salt stress increased electrical conductivity, osmotic pressure, Na^+^ accumulation, antioxidant enzyme activities, and proline content, while decreasing the K^+^/Na^+^ and Ca^2+^/Na^+^ ratios. Intracellular and extracellular polysaccharides showed distinct time-dependent accumulation patterns, and the corresponding crude extracts exhibited increased radical-scavenging activities under NaCl stress. Relative to the common 0 h baseline, RNA-seq identified 3654, 3843, 4289, 3566, and 2494 differentially expressed genes after 3, 6, 12, 24, and 48 h of NaCl exposure, respectively. These genes were enriched in pathways related to ribosomes, oxidative phosphorylation, protein processing in the endoplasmic reticulum, carbohydrate metabolism, and ABC transporters. *Gene05935*, encoding a predicted multi-pass transmembrane protein, showed consistently higher transcript abundance during NaCl exposure than at the common 0 h baseline. Heterologous expression of *Gene05935* in *Saccharomyces cerevisiae* was associated with higher mean endpoint OD_600_ values under the tested high-salinity conditions, while the *Arabidopsis thaliana* assay provided preliminary qualitative observations of seedling appearance during NaCl exposure. Because time-matched untreated controls were not included, the transcriptomic differences cannot be attributed specifically to NaCl. Together, the physiological measurements and temporal expression profiles identify *Gene05935* as a candidate for further functional characterization and may inform future cultivation-oriented evaluation of *Morchella* under saline conditions.

## 1. Introduction

*Morchella* spp. (Ascomycota, Pezizales, Morchellaceae) are highly valued edible and medicinally important fungi rich in proteins, polysaccharides, vitamins, and diverse bioactive compounds. Owing to their notable antioxidant, immunomodulatory, and antitumor activities, morels have become increasingly important in the global edible mushroom industry [1,2]. In recent years, the rapid expansion of commercial cultivation in China has made *Morchella* one of the most commercially promising edible fungi [3,4]. Understanding how *Morchella* mycelia respond to salinity may help assess the physiological constraints on cultivation under saline conditions.

Salinity imposes two related but distinct challenges: osmotic stress, resulting from reduced external water availability, and ionic stress, arising from excessive ion accumulation and disruption of intracellular ion balance [5,6,7]. Osmotic stress challenges cellular water balance and turgor, whereas ion toxicity can impair enzyme activity and membrane function. Both processes may contribute to oxidative stress [8], but antioxidant responses alone do not distinguish their respective contributions. Fungal adaptation to saline conditions involves osmotic adjustment, ion homeostasis, membrane remodeling, and stress-responsive signaling [6,9,10]. These processes provide a framework for investigating NaCl responses in *Morchella*, although their relative contributions cannot be resolved from NaCl treatment alone.

Studies in *Aspergillus nidulans* and yeasts have established important roles for osmotic-stress signaling and osmoadaptation [11,12]. In *Morchella*, previous work has examined the physiological responses of *M. sextelata* mycelia to NaCl and used principal component analysis to integrate the measured traits [13]. This provides a physiological basis for further investigation, but does not establish the causal roles of individual genes or polysaccharides in salt tolerance. How intracellular and extracellular polysaccharide dynamics relate to physiological responses and temporal transcriptional changes during NaCl exposure remains insufficiently resolved.

Polysaccharides are another relevant component of *Morchella* physiology. Antioxidant activity has been reported for polysaccharide preparations from *M. esculenta* [14], providing a rationale for examining intracellular polysaccharides (IPS) and extracellular polysaccharides (EPS) during NaCl exposure. However, antioxidant activity measured in extracted preparations does not establish that these compounds directly protect living *Morchella* mycelia from ROS under salt stress. Changes in IPS and EPS abundance and the radical-scavenging activities of their crude extracts should therefore be interpreted as associated responses rather than direct evidence of a protective mechanism.

RNA-seq enables the characterization of transcriptional changes during NaCl exposure [15], while weighted gene co-expression network analysis (WGCNA) can identify co-expressed gene modules associated with physiological traits [16]. Its application to *Sarcomyxa edulis* illustrates the use of this approach to prioritize genes associated with fungal biological processes [17]. In *M. sextelata*, combining physiological measurements with time-course transcriptomics may help identify candidate genes associated with polysaccharide dynamics and other stress-related traits. Such associations provide hypotheses for subsequent testing rather than evidence of causal regulation.

Heterologous expression provides a tractable approach for examining candidate genes in experimentally accessible hosts [18,19]. Here, *Saccharomyces cerevisiae* was used for quantitative endpoint growth comparisons across NaCl concentrations, whereas *Arabidopsis thaliana* was used for preliminary qualitative observation of seedling appearance during NaCl exposure. These assays provided different levels of phenotypic evidence for evaluating *Gene05935*, which encodes a predicted multi-pass transmembrane protein, with the plant observations serving as supplementary descriptive information.

For salt-tolerance research to inform morel cultivation, the reproductive potential of the selected isolates must also be considered. Given the importance of mating-type composition in morel reproduction [2,20], we focused on isolates in which both *MAT1-1* and *MAT1-2* idiomorphs were detected as a cultivation-oriented criterion for potential reproductive competence. This approach connects the investigation of vegetative salt tolerance with the practical goal of selecting materials for future cultivation trials.

Within this cultivation-oriented framework, we investigated the physiological and transcriptional responses of a selected *M. sextelata* isolate to NaCl exposure. We hypothesized that these responses would involve coordinated changes in ion balance, osmotic adjustment, antioxidant responses, and IPS/EPS dynamics. Physiological characterization, time-course transcriptomics, and WGCNA were integrated to explore these relationships, while heterologous expression of *Gene05935* was used to assess its potential contribution to salt-stress performance. The study aimed to provide a physiological and molecular basis for future evaluation and improvement of morel strains intended for cultivation under saline conditions.

## 2. Materials and Methods

### 2.1. Strain Collection and Identification

A total of 34 cultivated strains from China and 14 wild strains were collected (Table S1). All strains were isolated from tissue samples and preserved in the Fungal Culture Collection Room of Ludong University. Genomic DNA was extracted from actively growing mycelia, and the internal transcribed spacer (ITS) region was amplified using universal ITS primers. Polymerase chain reaction (PCR) products were visualized by agarose gel electrophoresis and subsequently sequenced. The resulting ITS sequences were compared against reference *Morchella* sequences in the NCBI database using BLAST (version 2.17.0+) to assess sequence similarity and taxonomic placement. ITS sequences from the 24 isolates retained for subsequent analyses were analysed together with reference sequences retrieved from GenBank using Bayesian inference, with *M. rufobrunnea* (GenBank accession KM485959) designated as the outgroup. Original isolate identifiers were retained throughout the study. Species names recorded at collection are distinguished from the taxonomic placements inferred from the ITS analysis presented here. Species assignments in this study are treated as ITS-based phylogenetic placements rather than multilocus-confirmed identifications.

### 2.2. Strain Revitalization and Purification

All strains were reactivated on potato dextrose agar (PDA). Mycelial plugs were transferred onto fresh PDA plates and incubated at 22 °C in darkness until the mycelium fully colonized the medium. The revitalized strains were then purified through repeated subculturing on PDA. After several rounds of transfer, isolates showing uniform colony morphology and no visible contamination were used for subsequent experiments. This operational assessment of culture purity did not establish genetic homogeneity or a single-spore origin.

### 2.3. Detection of Mating-Type Genes

Because successful fruiting in cultivated *Morchella* is generally associated with the coexistence of both mating-type idiomorphs within the cultivation system, detection of *MAT1-1* and *MAT1-2* was performed for all strains. Genomic DNA from all 48 strains was used as the template, and specific primers targeting the two mating-type genes (Table S2) were used for PCR amplification. The presence or absence of *MAT1-1* and *MAT1-2* amplicons was used to characterize the mating-type composition of each isolate.

PCR reactions (50 µL) consisted of: 25 µL 2× SanTaq Master Mix, 1 µL genomic DNA, 2 µL each primer, and 20 µL nuclease-free water. Thermal cycling conditions: 94 °C for 3 min; 35 cycles of 94 °C for 30 s, 55 °C for 50 s, 72 °C for 90 s; and a final extension at 72 °C for 10 min. Amplicons were separated on 1% agarose gels in 1× TAE buffer at 100 V for 30 min. DNA marker (6 µL) and PCR products (6 µL) were loaded, and bands were visualized under UV illumination. Strains in which both *MAT1-1* and *MAT1-2* were detected were retained for further study.

### 2.4. Assessment of Growth Traits and Calculation of Composite Scores

Many fungal stress-response traits, such as growth rate, mycelial density, and sclerotia formation, are biologically coupled; therefore, direct averaging of these indicators would overestimate the contribution of correlated traits. PCA summarizes variation among correlated traits using orthogonal components [21]. Here, PCA-informed grouping and membership-function analysis were used to construct a composite index for comparing growth-related performance under the specified NaCl screening conditions [22].

The salt tolerance of different strains was initially evaluated by culturing them on PDA solid medium supplemented with 1 mol·L^−1^ NaCl. This concentration was used solely as a stringent screening threshold to accentuate phenotypic differences among isolates, consistent with previous in vitro screening of salt-tolerant fungi at NaCl concentrations of up to 1 mol·L^−1^ [23]. Colony growth characteristics and mycelial physiological parameters were measured and recorded. The scoring criteria for mycelial germination score, mycelial density, and sclerotia number were defined according to Table S3, Table S4 and Table S5, respectively. For the revised ranking, two grouped indices were reconstructed from the rounded individual trait coefficients (c) reported in Table S7 and converted into membership values using min–max normalization across the 24 isolates. These membership values were then combined using normalized variance-contribution weights to obtain the composite screening score:

*CI*_1*i*_ = *c_i_*_,MD_ + *c_i_*_,SN_ + *c_i_*_,FW_ + *c_i_*_,DW_

*CI*_2*i*_ = *c_i_*_,GR_ + *c_i_*_,FWC_ + *c_i_*_,GT_

$$u_{\mathrm{ki}}=\frac{{\mathrm{CI}}_{\mathrm{ki}}-{\mathrm{CI}}_{k,\min}}{{\mathrm{CI}}_{k,\max}-{\mathrm{CI}}_{k,\min}},k=1,2$$

$$D_{i}=\frac{60.29}{75.73}u_{1i}+\frac{15.44}{75.73}u_{2i}\approx 0.7961u_{1i}+0.2039u_{2i}$$

### 2.5. Salt Stress Gradient Screening

To determine the selected NaCl concentration for salt-stress treatment, PDA medium was supplemented with six NaCl concentrations (0, 100, 200, 300, 400, and 500 mmol·L^−1^). Mycelial plugs of identical growth status were inoculated centrally onto each plate and incubated at 22 ± 1 °C in darkness.

Mycelia were inoculated onto PDA plates covered with sterile cellophane membranes (0.05 mm thick; purchased from Beijing Tsingke Biotech Co., Ltd., Beijing, China) and cultured for 48 h until stable growth was established. The cellophane membranes carrying actively growing mycelia were then transferred onto PDA plates containing different concentrations of NaCl for salt-stress treatment. After treatment, the PDA cultures were used to determine mycelial growth rate, electrical conductivity, malondialdehyde (MDA) content, osmotic pressure, soluble protein content, free proline content, SOD, POD, and CAT activities. Ion contents were measured using mycelia grown on PDA solid medium as described in Section 2.6.1. In contrast, IPS and EPS were determined using liquid cultures supplemented with the corresponding NaCl concentrations, as described in Section 2.6.2.

Hyphal diameter was measured using three independently cultured plates as biological replicates for each treatment. A drop of sterile distilled water was first placed on a clean glass slide. For each replicate plate, hyphae were collected from five sampling positions (top, bottom, left, right, and center), gently transferred into the water drop, and covered with a coverslip. The coverslip was lightly tapped to disperse the hyphae as evenly as possible. Hyphae were first located under 40× magnification using a metallographic microscope (OLYMPUS BX61; Olympus Corporation, Tokyo, Japan), and their diameters were subsequently measured under 100× magnification. A total of 100 hyphae were measured per treatment, with 33–34 hyphae randomly measured from each replicate plate and distributed across the five sampling positions. Measurements of individual hyphae were considered subsamples rather than independent biological replicates. The mean hyphal diameter was calculated separately for each plate, and the three plate-level means were used for statistical analysis (*n* = 3).

After selecting the representative NaCl concentration, sampling time points of 0, 3, 6, 12, 24, and 48 h were selected based on the growth characteristics of *Morchella* and preliminary experimental observations.

### 2.6. Determination of Physiological and Biochemical Parameters

#### 2.6.1. Physiological and Biochemical Indicators of Stress Resistance

Before physiological and ion-content measurements, harvested mycelia were rapidly rinsed with sterile distilled water to remove surface-adhered NaCl, gently blotted dry with filter paper, and immediately used for subsequent analyses.

(1) Conductivity and MDA content: For electrical conductivity measurements, 1.0 g of fresh mycelia from each biological replicate was collected after NaCl treatment, rinsed three times with deionized water to remove surface-adhered salts, gently blotted dry, and transferred to a 100 mL Erlenmeyer flask containing 40 mL of bidistilled water. The samples were incubated at 25 °C for 2 h, after which the electrical conductivity of the mycelial leachate was measured using an MP515-02 conductivity meter (Shanghai San-Xin Instrumentation, Inc., Shanghai, China). The results were expressed as absolute electrical conductivity (mS·cm^−1^) [24], and mycelial MDA content was determined using the thiobarbituric acid (TBA) method [25].
(2) Osmotic adjustment substances: free proline was determined by the acid ninhydrin colorimetric method; soluble protein was detected by the Coomassie Brilliant Blue G-250 method [26].
(3) Antioxidant enzyme system: CAT activity was measured by the H_2_O_2_ decomposition rate method; POD activity was determined by the guaiacol method; SOD activity was determined by the nitroblue tetrazolium (NBT) photoreduction inhibition rate method [27,28].
(4) Na, K, and Ca were measured by inductively coupled plasma atomic emission spectrometry (ICP-AES), with additional dilution where necessary, and contents were expressed as mg·g^−1^ dry weight. K^+^/Na^+^ and Ca^2+^/Na^+^ mass ratios were calculated for the KCl- and CaCl_2_-supplemented experiments, respectively.

#### 2.6.2. Extraction and Quantification of Intracellular and Extracellular Polysaccharides

Liquid culture was used for IPS and EPS determination because it facilitated the collection of fermentation broth and the extraction and quantification of intracellular and extracellular polysaccharides. For polysaccharide extraction, mycelia were cultured for 5 days in liquid medium supplemented with the corresponding NaCl concentrations under the same temperature conditions described above. The cultures were filtered through a 140-mesh nylon filter to separate the fermentation broth from the mycelia. The fermentation broth was used for extracellular polysaccharide (EPS) extraction. The filtered mycelia were washed 2–3 times with distilled water to remove residual NaCl and then dried at 53 °C to constant weight.

EPS were extracted from the fermentation broth by ethanol precipitation (four volumes of 95% ethanol, 4 °C for 24 h), followed by centrifugation. Intracellular polysaccharides (IPS) were extracted from dried mycelial powder using an ultrasound-assisted hot-water extraction method and subsequently precipitated with ethanol as described above. Polysaccharide contents were determined using the phenol–sulfuric acid method, with absorbance measured at 490 nm and glucose used as the standard [29].

#### 2.6.3. Antioxidant Activity of Crude Polysaccharide Extracts

Hydroxyl radical (•OH) scavenging activity was assessed using the Fenton reaction (H_2_O_2_–Fe^2+^ system), with absorbance measured at 510 nm [30]. DPPH radical scavenging activity was determined by measuring the absorbance at 517 nm [31]. Reducing power was evaluated using the potassium ferricyanide reduction method, with absorbance measured at 700 nm after incubation at 50 °C.

### 2.7. Transcriptome Sequencing and Quantitative Reverse Transcription PCR (qRT-PCR) Validation

All cultures were pre-cultured for 48 h under identical conditions. NaCl treatments were then initiated in reverse chronological order so that samples exposed to NaCl for 0, 3, 6, 12, 24, and 48 h were harvested simultaneously, thereby minimizing variation associated with culture batch and sampling time. Samples collected at 0 h, immediately before NaCl treatment, were used as the common reference for the 3, 6, 12, 24, and 48 h samples. Each comparison therefore assessed transcriptional changes during NaCl exposure relative to the initial baseline. Three independent biological replicates were prepared for each time point. No time-matched untreated controls were included in the RNA-seq experiment. Therefore, the comparisons capture temporal transcriptional differences during NaCl exposure relative to the initial 0 h baseline and cannot distinguish NaCl-specific effects from cultivation-related temporal changes.

Total RNA was extracted from each sample, and RNA integrity and purity were evaluated before library construction. RNA-seq library construction and high-throughput sequencing were performed by Guangzhou Genedenovo Biotechnology Co., Ltd. (Guangzhou, China). Subsequent in-depth bioinformatic reanalysis was performed by Tsingke Biotechnology. Paired-end sequencing was used [15]. The raw-base and raw-read totals in the sequencing report indicate a read length of 150 nt. Reads were quality-filtered using fastp v0.20.0. The reported filtering criteria excluded reads shorter than 100 nt after adapter and terminal low-quality trimming, reads containing more than 10 undetermined bases, and reads with more than 40% of bases having quality scores ≤ 30. Clean reads were aligned to the *M. sextelata* reference genome (GenBank assembly accession ASM974175v1) using HISAT2 v2.2.1. BAM files were processed using SAMtools v1.10, and RNA-seq quality assessment used RSeQC v2.6.4. Transcript reconstruction and expression quantification used StringTie v2.0.4, with FPKM and TPM values reported. The raw RNA-seq data generated in this study have been deposited in the NCBI Sequence Read Archive under BioProject accession number PRJNA1465190.

Differentially expressed genes (DEGs) were identified using DESeq2 v1.26.0 [32]. DEGs were defined by an adjusted *p* value (padj) < 0.05 and |log_2_FoldChange| ≥ 1. Functional annotation and enrichment analyses used the Gene Ontology (GO) and Kyoto Encyclopedia of Genes and Genomes (KEGG) databases with clusterProfiler v3.14.0. The sequencing report specified genome-based background sets and a nominal *p* ≤ 0.05 threshold for GO and KEGG enrichment, with a hypergeometric test specified for KEGG. Nominal enrichment *p* values are distinct from the adjusted *p* values used for DEG selection; the enrichment results were interpreted descriptively.

Twelve representative DEGs with different expression patterns were selected for qRT-PCR validation (Table S6). Total RNA was extracted using the RNAprep Pure Plant Kit (Vazyme, Nanjing, China), quantified using a NanoDrop spectrophotometer (Thermo Fisher Scientific, Wilmington, DE, USA), and reverse-transcribed using the HiScript II 1st Strand cDNA Synthesis Kit (Vazyme, Nanjing, China). Primers (yielding 100–200 bp products) were designed using Primer-BLAST and synthesized by Sangon Biotech (Shanghai, China).

qRT-PCR reactions (20 µL) contained 10 µL of 2× ChamQ SYBR Master Mix (Vazyme, Nanjing, China), 1 µL of cDNA, 0.4 µL of each primer, and 8.2 µL of ddH_2_O. Cycling conditions were 95 °C for 30 s, followed by 40 cycles of 95 °C for 10 s and 60 °C for 30 s.

Reactions were performed on a StepOne Plus Real-Time PCR System (ABI, CA, USA). *ACT1* served as the internal reference, and relative expression levels were calculated using the 2^−ΔΔCt^ method [33]. Data are presented as mean ± standard deviation (SD) from three independent biological replicates. Differences were considered statistically significant at *p* < 0.05.

### 2.8. Methods for Weighted Gene Co-Expression Network Analysis

#### 2.8.1. Network Construction

FPKM-normalized expression values from 18 RNA-seq libraries were used for WGCNA [16,34]. The reported network contained 8249 genes after expression and variability filtering. A soft-thresholding power of 24 was selected based on a scale-free topology fit index above 0.85, and modules were identified using the dynamic tree cut method with default parameters [16].

#### 2.8.2. Exploratory Module–Trait Analysis and KEGG Pathway Enrichment Analysis

Ten physiological traits were measured using three independent biological replicates at each of six time points. Physiological measurements and RNA sequencing were performed using two separately prepared sets of cultures under the same cultivation conditions. Paired cultures were established at the start of the experiment, with one member of each pair used for physiological measurements and the other for RNA sequencing. The 18 rows of the physiological trait matrix were aligned with the RNA-seq samples by time point and replicate identity according to the original pairing records. Each pair therefore comprised two distinct cultures. The traits included hyphal diameter (HD), dry weight (DW), intracellular polysaccharide (IPS) content, extracellular polysaccharide (EPS) content, IPS-hydroxyl radical scavenging activity (IPS-HRSA), EPS-hydroxyl radical scavenging activity (EPS-HRSA), IPS-DPPH radical scavenging rate (IPS-DPPH-SR), EPS-DPPH radical scavenging rate (EPS-DPPH-SR), IPS-reducing power (IPS-RP), and EPS-reducing power (EPS-RP).

Module eigengenes were calculated from the network constructed using all 18 RNA-seq libraries. Pearson correlations were used to describe module–trait relationships, as reported in Figure S10. The displayed *p* values are consistent with conventional two-sided Pearson tests using 18 observations; these are nominal *p* values, and no Benjamini–Hochberg correction was performed across module–trait tests. Although the two culture sets were paired by experimental design, the molecular and physiological measurements were obtained from different cultures. The correlations were therefore interpreted as exploratory associations across the paired time-course samples and may also reflect shared temporal trends.

The lightcyan, brown, darkred, grey60, and darkslateblue modules were selected for follow-up because they showed relatively high absolute correlations with the measured traits. Selection was exploratory and was not based on significance after multiple-testing correction. KEGG pathway enrichment analyses of these modules were used to describe their functional composition. These enrichment results were interpreted descriptively and were not considered independent evidence that the modules regulate the measured physiological traits.

### 2.9. Functional Annotation and Heterologous Expression of a Candidate Gene Identified During NaCl Exposure

#### 2.9.1. Bioinformatic Characterization of the Candidate Protein

Physicochemical properties of candidate proteins were analyzed using ProtParam (for molecular weight and isoelectric point) and ProtScale (for hydrophobicity) [35], SOPMA (for secondary structure), and TMHMM (for transmembrane region prediction) [36]. Conserved domains were predicted using InterPro.

#### 2.9.2. Heterologous Expression and Qualitative Seedling Assessment in *A. thaliana*

Wild-type *Arabidopsis thaliana* Columbia-0 (Col-0) was used for heterologous expression analysis. The *Gene05935* coding sequence was inserted into pBI121 between the XbaI and SacI sites, under the control of the CaMV 35S promoter and NOS terminator. After sequence verification, the recombinant construct was introduced into *Agrobacterium tumefaciens* GV3101 and transformed into Col-0 plants using the floral-dip method. The *Agrobacterium* culture was grown to an OD_600_ of 0.8 before transformation [18]. Seeds obtained following transformation were selected on half-strength MS medium containing kanamycin, and plants were propagated to the T3 generation before the plate assay. Three independent transformation events were obtained. Plants carrying empty pBI121 served as controls. Seedlings with similar initial growth were exposed to 100, 200, or 300 mmol·L^−1^ NaCl for 15 days. Three replicate sets were established, each containing one plate at each of the three NaCl concentrations, giving nine plates and 18 seedlings in total. Each plate contained two seedlings: one empty-vector control and one *Gene05935*-transgenic seedling, grown under shared culture conditions. Each plate constituted one replicate for the qualitative comparison. Seedling appearance was assessed visually with knowledge of plant identities, and representative photographs were selected for presentation. No quantitative phenotypic endpoints were measured or subjected to inferential statistical analysis. The assay was therefore used for preliminary qualitative observation rather than functional validation of enhanced salt tolerance. *Gene05935* transcripts were assessed by qRT-PCR with *ACT1* as the internal reference. Amplification and melting-curve outputs were examined, with late control signals interpreted cautiously because their product identity was not established.

#### 2.9.3. Heterologous Expression of *M. sextelata Gene05935* in *Saccharomyces cerevisiae* Under Salt Stress

Total RNA was extracted from *M. sextelata* mycelia and reverse-transcribed into cDNA as described above. The *Gene05935* coding sequence was amplified, cloned into pYES2, and verified by sequencing after transformation into *Escherichia coli* DH5α. The confirmed construct was introduced into *Saccharomyces cerevisiae* INVSc1, with the empty pYES2 vector used as the control. Transformants were selected and cultured in SC-Ura medium containing 1% galactose. Cultures were adjusted to an initial OD_600_ of 1.0 and serially diluted tenfold to give the five spotting dilutions 10^0^, 10^−1^, 10^−2^, 10^−3^, and 10^−4^. Aliquots of 5 μL were spotted onto SC-Ura agar plates containing 0, 0.5, 1.0, or 1.5 mol·L^−1^ NaCl and incubated at 30 °C for 5 days. The spotting assay was independently repeated three times. For the liquid-culture assay, *S. cerevisiae* INVSc1 carrying pYES2-*Gene05935* or empty pYES2 was precultured, and the cultures were adjusted to the same OD_600_ before NaCl treatment. Both strains were incubated under identical selection and induction conditions in SC-Ura liquid medium containing 1% galactose at NaCl concentrations of 0, 0.5, 1.0, 1.5, 2.0, and 2.5 mol·L^−1^. The liquid assay extended the concentration range to assess 24 h endpoint growth at higher salinity. Endpoint OD_600_ was measured after 24 h of incubation at 30 °C. Three independent biological replicates were used for each construct–concentration combination, with three technical readings averaged for each biological replicate. Results were expressed as mean ± SD. *Gene05935* transcripts were assessed by qRT-PCR with *ACT1* as the internal reference. Amplification and melting-curve outputs were examined, with late control signals interpreted cautiously because their product identity was not established.

### 2.10. Statistical Analysis

Unless otherwise specified, experimental data are presented as mean ± standard deviation (SD) from three independent biological replicates (*n* = 3). Statistical analyses were performed using SPSS 19.0. For concentration-gradient experiments evaluated at a single endpoint, differences among NaCl concentrations were assessed using one-way analysis of variance (ANOVA), followed by Tukey’s post hoc test, including the comparisons of ion contents. For the time-course data in Figure 3, separate one-way ANOVAs were performed at each time point to compare the control and NaCl treatment, followed by Tukey’s test. These comparisons assessed treatment differences at individual time points; the treatment-by-time interaction was not tested. Tukey’s adjustment applied within each analysis, with no additional correction across time points. For hyphal diameter, individual hyphae were treated as subsamples, and the mean for each independently cultured plate was used for statistical analysis. Where technical readings were obtained, they were averaged within each biological replicate before analysis. Differences were considered statistically significant at *p* < 0.05. Figures were prepared using SigmaPlot 10.0. Principal component analysis (PCA) was used to summarize variation in physiological profiles across NaCl concentrations and to visualize the relationships among measured traits. For each concentration-level PCA, the input comprised 18 observations representing three independent biological replicates at each of six NaCl concentrations, with one replicate per row and one physiological variable per column. Before analysis, each variable was Z-score standardized by subtracting its mean across the 18 observations and dividing by its sample standard deviation. The analyses served as descriptive complements to the comparisons of individual physiological indicators. The *Arabidopsis* seedling photographs were evaluated qualitatively and were not included in the quantitative phenotypic analyses or significance testing. Two concentration PCA analyses were performed separately, one without EPS and the other with EPS. Their explained-variance percentages refer to their respective analyses and are not interchangeable.

## 3. Results

### 3.1. Strain Identification and Detection of Mating-Type Genes

PCR amplification of *MAT1-1* and *MAT1-2* showed that 24 isolates yielded amplicons for both idiomorphs (Figure S1), which is consistent with the mating-type complexity reported in cultivated *Morchella* spp. The strains carrying both *MAT1-1* and *MAT1-2* idiomorphs included Nos. 1, 2, 7–15, 21–23, 27, 29, 31, 35, 36, 38–40, 45, and 46. Detection of both mating-type idiomorphs was used as a cultivation-oriented molecular preselection criterion. Bayesian phylogenetic analysis based on ITS sequences divided the 24 selected isolates into three major clades. Strains 35 and 38 clustered with reference sequences of *M. crassipes*, strains 36 and 45 clustered with *M. esculenta*, strains 1, 13, 27, and 31 clustered with *M. importuna*, and the remaining 16 isolates, including strain 21, clustered with *M. sextelata* (Figure S2). Detection of both idiomorphs in these tissue-derived cultures does not by itself establish their nuclear composition, genetic homogeneity, or fruiting ability.

### 3.2. Comprehensive Evaluation of Growth-Related Traits

The 24 preselected isolates, representing *M. sextelata*, *M. importuna*, *M. esculenta*, and *M. crassipes*, were evaluated on PDA medium containing 1 mol·L^−1^ NaCl. Significant differences (*p* < 0.05) were observed among strains in mycelial growth rate, density, sclerotial formation, and dry weight. Salt-tolerance coefficients for individual traits are presented in Table S7.

Correlation analysis (Table S8) showed that most growth-related traits were positively correlated. Growth rate was significantly associated with mycelial density, sclerotia number, fresh and dry weight, fresh water content, and germination score. PC1 and PC2 explained 60.29% and 15.44% of the total variance, respectively, with a cumulative contribution of 75.73% (Table S9). PC1 was mainly related to dry weight, fresh weight, and mycelial density, whereas PC2 was associated with germination score, growth rate, and fresh water content. Their normalized weights in the composite score were 79.61% and 20.39%, respectively.

Using the rounded trait coefficients reported in Table S7 and the stated PCA-informed grouping and membership-function procedure, Mel-21 obtained the highest reconstructed composite screening score (*D* = 0.94926) among the 24 isolates under 1 mol·L^−1^ NaCl. Isolate 8 had a reconstructed *D* value of 0.35456 and ranked 17th. Isolates 35 and 45 both had *D* = 0 and were assigned the shared rank of 23 (Table 1). Closely spaced ranks should be interpreted cautiously because the source coefficients were rounded.

### 3.3. Screening of Salt Stress Concentration and Physiological Response of M. sextelata Strain Mel-21

#### 3.3.1. Effects of NaCl Stress on Mycelial Growth and Morphology

**Figure 1:**
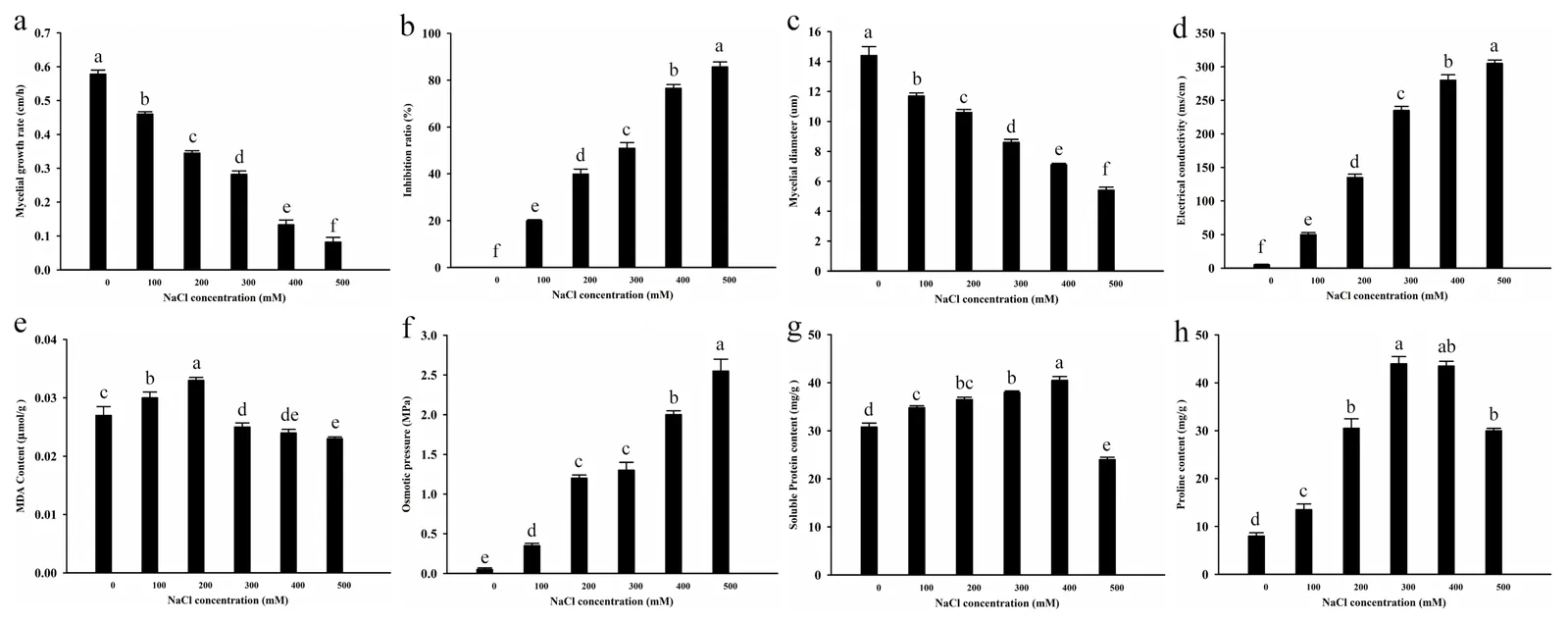
Effects of different NaCl concentrations on the growth and physiological responses of *M. sextelata* mycelia. (**a**) Mycelial growth rate; (**b**) growth inhibition rate; (**c**) hyphal diameter; (**d**) electrical conductivity; (**e**) malondialdehyde (MDA) content; (**f**) osmotic pressure; (**g**) soluble protein content; and (**h**) proline content. Note: Following a 2-day preculture, mycelia were transferred to PDA containing the indicated NaCl concentrations, and all treatments were sampled when the control colonies fully covered the plates. Bars represent means ± SD from three independently cultured plates per treatment (*n* = 3). For (**c**), 100 hyphae per treatment (33–34 per plate) were measured as subsamples; statistical analysis used the three plate-level means. Different lowercase letters indicate significant differences among NaCl concentrations within each panel (*p* < 0.05; one-way ANOVA followed by Tukey’s test).

*M. sextelata* mycelia germinated under all tested NaCl concentrations (Figure S3), but colony development and growth rate decreased with increasing salinity. Within 12 h, the control, 100 mmol·L^−1^, and 200 mmol·L^−1^ treatments showed normal germination with dense white mycelia, with mean growth rates of 0.58, 0.46, and 0.34 cm·d^−1^, respectively (Figure 1a). Germination was delayed to 24 h under 300 and 400 mmol·L^−1^ NaCl, with growth rates of 0.28 and 0.13 cm·d^−1^, and further delayed to 48 h under 500 mmol·L^−1^, where mycelia were sparse and weak. The inhibition rate increased progressively and reached the maximum at 500 mmol·L^−1^ (Figure 1b).

Increasing NaCl concentration also reduced hyphal diameter (Figure S4). The control group showed the largest hyphal diameter (14.45 μm), whereas diameters decreased to 11.64 and 10.63 μm under 100 and 200 mmol·L^−1^ NaCl, respectively (Figure 1c). A more pronounced reduction was observed above 200 mmol·L^−1^, and the diameter reached 5.43 μm at 500 mmol·L^−1^. The control value was approximately 2.66-fold higher than that at 500 mmol·L^−1^ (*p* < 0.05; Figure 1c).

Mycelial biomass showed a similar decreasing trend (Table 2). Both fresh weight and dry weight decreased with increasing salinity. Compared with the control group, biomass in the 100–300 mmol·L^−1^ treatments was significantly lower (*p* < 0.05), whereas no significant differences were detected among the 300–500 mmol·L^−1^ treatments. The water content of fresh mycelia remained relatively stable under 0–200 mmol·L^−1^ NaCl, reaching 95.79% at 200 mmol·L^−1^, and then decreased to 91.58% at 500 mmol·L^−1^.

#### 3.3.2. Effects of NaCl Stress on Membrane Damage and Osmotic Regulation

Electrical conductivity increased markedly with increasing NaCl concentration. Conductivity rose from 4.76 mS·cm^−1^ in the control group to 310.87 mS·cm^−1^ at 500 mmol·L^−1^ NaCl, representing a 65.31-fold increase (Figure 1d). The most pronounced increase occurred between 200 and 300 mmol·L^−1^ (*p* < 0.05). MDA content initially increased, reaching a maximum value of 0.033 μmol·g^−1^ at 200 mmol·L^−1^ NaCl (*p* < 0.05; Figure 1e), and then declined to 0.006 μmol·g^−1^ at 500 mmol·L^−1^. Osmotic pressure also increased with salinity, rising from 0.06 MPa in the control group to 2.78 MPa at 300 mmol·L^−1^ NaCl (Figure 1f).

Osmotic adjustment-related substances were also altered by NaCl stress. Soluble protein and proline contents showed significant changes under salinity stress. Soluble protein content increased under moderate salt stress and reached a maximum value of 40.47 mg·g^−1^ at 400 mmol·L^−1^ NaCl, which was significantly higher than that of the control group (*p* < 0.05; Figure 1g), and then decreased at higher salinity levels. Proline content increased sharply under NaCl stress, showing a 61.44–434.31% increase compared with the control group at 100–300 mmol·L^−1^ NaCl (Figure 1h). The maximum proline content was 43.92 μg·g^−1^ at 300 mmol·L^−1^ NaCl, followed by a decline at higher salinity.

#### 3.3.3. Effects of NaCl Stress on Antioxidant Enzyme Activities and Ion Homeostasis

**Figure 2:**
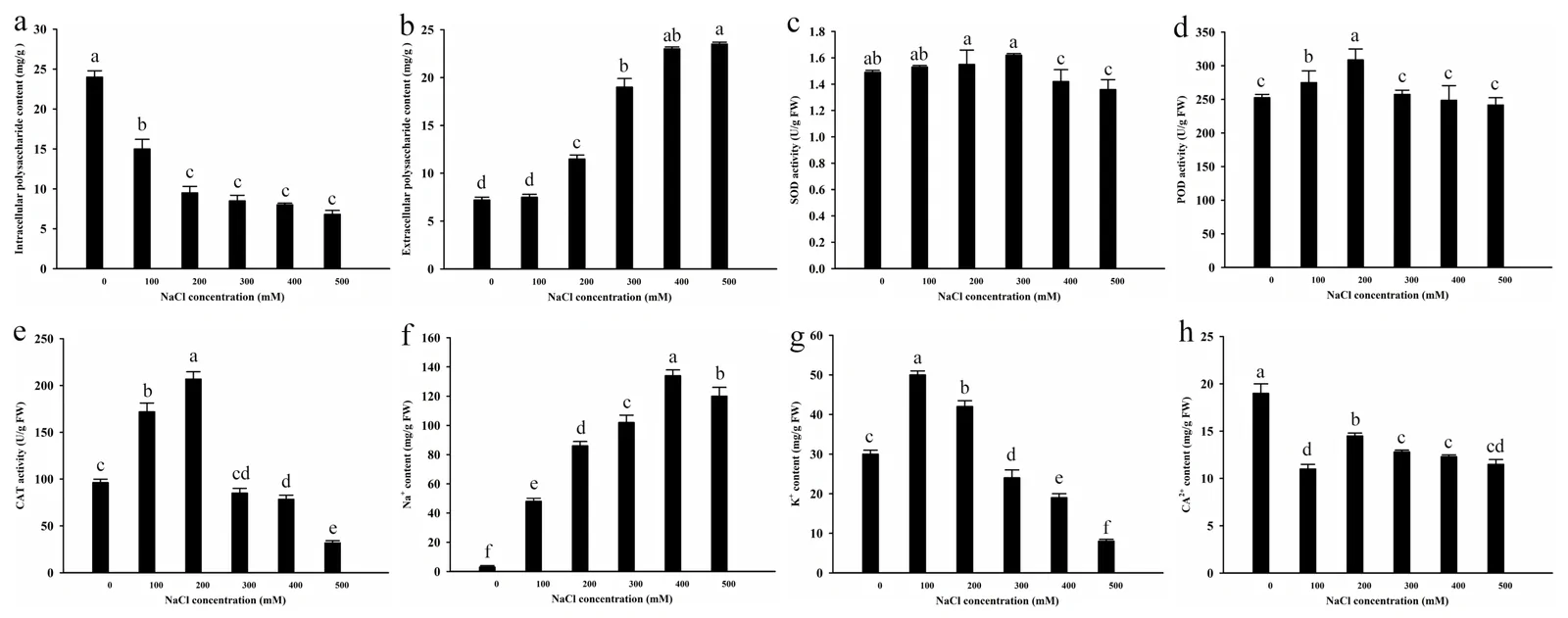
Effects of NaCl concentrations on polysaccharide contents, antioxidant enzyme activities, and ion contents in *M. sextelata* mycelia. Note: (**a**) Intracellular polysaccharide (IPS) content; (**b**) extracellular polysaccharide (EPS) content; (**c**) superoxide dismutase (SOD) activity; (**d**) peroxidase (POD) activity; (**e**) catalase (CAT) activity; (**f**) Na^+^ content; (**g**) K^+^ content; and (**h**) Ca^2+^ content. Bars represent means ± SD from three independent cultures per treatment (*n* = 3). For (**a**,**b**), biological replicates were independent liquid cultures grown for 5 days at the indicated NaCl concentrations. For (**c**–**h**), biological replicates were independently cultured PDA plates; following a 2-day preculture, NaCl treatments were initiated simultaneously and sampled when the control colonies fully covered the plates. Statistical analyses used independent culture-level observations. Different lowercase letters indicate significant differences among NaCl concentrations within each panel (*p* < 0.05; one-way ANOVA followed by Tukey’s test).

IPS and EPS contents were affected by NaCl stress. IPS content decreased continuously with increasing NaCl concentration (Figure 2a), whereas EPS content increased progressively under low to moderate salinity and declined at higher concentrations (Figure 2b).

Antioxidant enzyme activities showed distinct responses to increasing NaCl concentrations. SOD activity increased gradually with increasing salinity and reached its maximum at 300 mmol·L^−1^ NaCl, showing an 8.72% increase compared with the control group, and then declined sharply at higher salt concentrations (Figure 2c). POD activity increased markedly at 100 mmol·L^−1^ NaCl and peaked at 200 mmol·L^−1^ NaCl with a maximum activity of 308.75 U·g^−1^, after which it decreased continuously with further increases in NaCl concentration (Figure 2d). CAT activity showed a similar pattern and reached its highest value at 200 mmol·L^−1^ NaCl (206.88 U·g^−1^), followed by a significant decline when the NaCl concentration exceeded 300 mmol·L^−1^ (Figure 2e).

The contents of Na^+^, K^+^, and Ca^2+^ in mycelia changed significantly under NaCl stress. Na^+^ content increased with increasing salinity and reached a maximum of 134.13 mg·g^−1^ at 400 mmol·L^−1^ NaCl, followed by a slight decrease at 500 mmol·L^−1^ (Figure 2f). In contrast, K^+^ content decreased continuously, from 50.65 mg·g^−1^ at 100 mmol·L^−1^ NaCl to 8.50 mg·g^−1^ at 500 mmol·L^−1^ (Figure 2g). Ca^2+^ content decreased under all NaCl treatments compared with the control, reaching the lowest value at 100 mmol·L^−1^ (Figure 2h).

In cultures supplemented with 20 mmol·L^−1^ KCl, the K^+^/Na^+^ mass ratio decreased progressively from 63.56 at 0 mmol·L^−1^ NaCl to 0.07 at 500 mmol·L^−1^ NaCl (Table S10). In the separate experiment supplemented with 20 mmol·L^−1^ CaCl_2_, the Ca^2+^/Na^+^ mass ratio decreased from 11.99 to 0.04 over the same NaCl concentration range (Table S11).

#### 3.3.4. Integrated Analysis of Physiological Responses Under NaCl Stress

A PCA using the physiological variables listed in Table 3, excluding EPS, was performed to summarize responses of *M. sextelata* mycelia under different NaCl concentrations. PC1 and PC2 explained 66.237% and 22.347% of the total variance, respectively. PC1 was mainly associated with mycelial growth rate, hyphal diameter, fresh weight, dry weight, and IPS content on the positive side, and with inhibition rate, electrical conductivity, and osmotic pressure on the negative side. PC2 was mainly associated with POD, CAT, and SOD activities, as well as MDA, soluble protein, and proline contents. The PCA provided an overview of the physiological differences among NaCl treatments. At 300 mmol·L^−1^ NaCl, the observed mean mycelial growth rate was reduced by 51.32% relative to the control while measurable growth was maintained, supporting the selection of this concentration for subsequent time-course experiments.

In the separately generated EPS-inclusive PCA biplot (Figure S5; PC1 = 71.1%, PC2 = 20.1%), the 0 and 100 mmol·L^−1^ NaCl treatments were distributed on the positive side of PC1 and were closely associated with growth-related traits, including growth rate, fresh weight, dry weight, and IPS content. The 400 and 500 mmol·L^−1^ treatments were located on the negative side of PC1 and were closely associated with electrical conductivity, inhibition rate, osmotic pressure, and EPS content. The 200 mmol·L^−1^ treatment was clearly separated along PC2 and was mainly associated with MDA content and antioxidant enzyme activities, especially POD and CAT. The 300 mmol·L^−1^ treatment occupied an intermediate position in the PCA plot. Its position distinguished it from the growth-associated 0–100 mmol·L^−1^ treatments, the antioxidant-associated 200 mmol·L^−1^ treatment, and the membrane- and osmotic-stress-associated 400–500 mmol·L^−1^ treatments.

### 3.4. Time-Dependent Responses of M. sextelata Strain Mel-21 at 300 mmol·L^−1^ NaCl

#### 3.4.1. Effects of Salt Stress on Mycelial Growth and Morphology

The responses of *M. sextelata* mycelia to 300 mmol·L^−1^ NaCl were time-dependent. Microscopic observation showed that hyphal diameter decreased progressively with increasing stress duration, whereas the overall hyphal morphology remained largely unchanged. The mycelia maintained a loose arrangement, and no obvious abnormalities such as severe twisting, swelling, or fragmentation were observed throughout the treatment period (Figure S6). Quantitative measurements further showed that hyphal diameter decreased significantly with prolonged salt stress. A significant difference was observed between 3 h and 6 h, and the 48 h treatment differed significantly from all other time points (Figure S7).

Mycelial dry weight increased over time in both the control and salt-treatment groups. At 3 h and 6 h, the dry weight of the salt-treated group was slightly but not markedly higher than that of the control group, possibly reflecting an early osmotic adjustment response rather than a true increase in biomass accumulation. However, with prolonged treatment, the dry weight of the salt-treatment group became lower than that of the control group and remained lower thereafter (Figure S8).

#### 3.4.2. Effects of Salt Stress on IPS and EPS Contents in Mycelia

**Figure 3:**
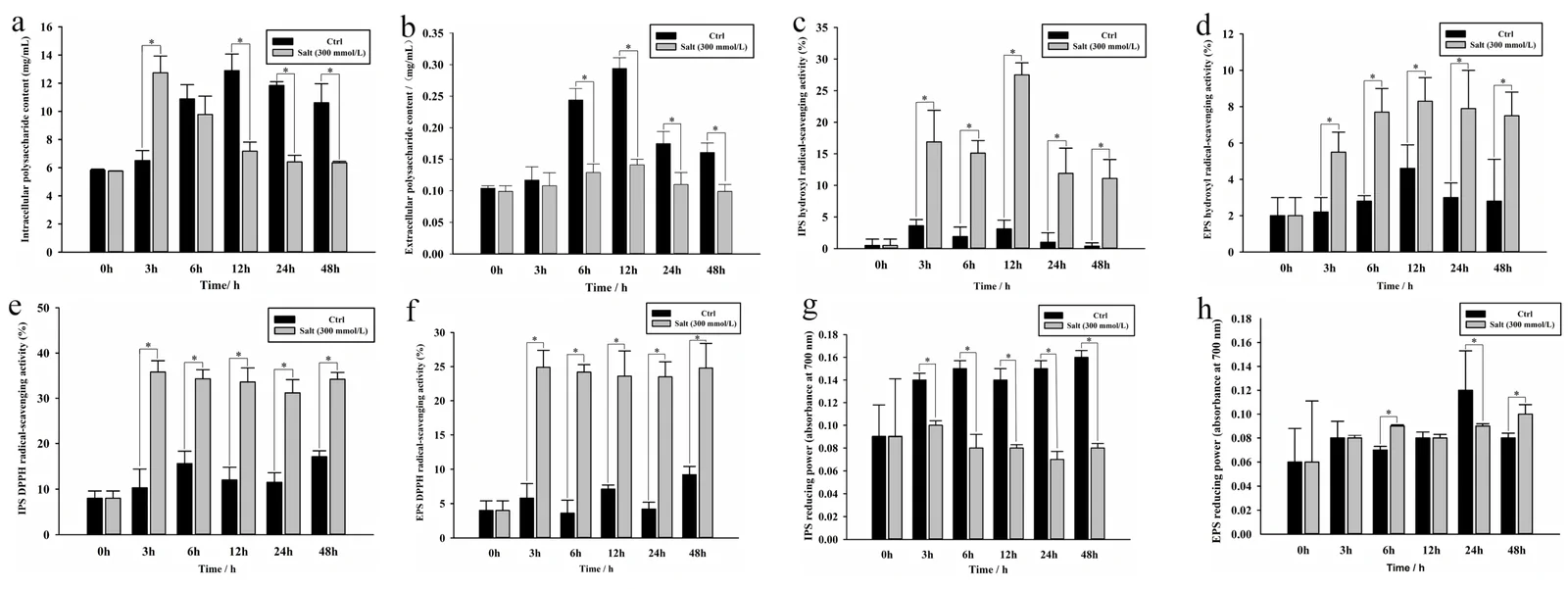
Time-dependent effects of 300 mmol·L^−1^ NaCl on polysaccharide contents and antioxidant activities in *M. sextelata* Mel-21 mycelia. (**a**,**b**) IPS and EPS contents; (**c**,**d**) hydroxyl radical-scavenging activity; (**e**,**f**) DPPH radical-scavenging activity; and (**g**,**h**) reducing power. In each panel pair, IPS precedes EPS. Antioxidant assays used crude IPS/EPS extracts. Data are means ± SD (*n* = 3 independent biological replicates). Asterisks indicate significant differences between the control and NaCl treatment at the same time point (*p* < 0.05; separate one-way ANOVAs followed by Tukey’s test).

The IPS and EPS contents of *M. sextelata* mycelia showed different temporal response patterns under 300 mmol·L^−1^ NaCl stress. In the control group, IPS content increased initially and then declined, reaching its maximum at 12 h. Under salt stress, however, IPS content peaked at 3 h and was significantly higher than that of the control group at this time point (Figure 3a). Thereafter, IPS content decreased progressively and fell below the control level with prolonged treatment.

EPS content in both the control and salt-treatment groups increased initially and then declined over time, reaching peak values at 12 h. However, EPS content in the salt-treatment group remained lower than that in the control group throughout the experiment and showed only slight variation with stress duration (Figure 3b).

#### 3.4.3. Effects of Salt Stress on Antioxidant Activities of Crude IPS and EPS Extracts

The •OH radical-scavenging activity of IPS in the salt-treatment group was higher than that in the control group at all time points (Figure 3c). In the control group, the values at 3, 6, 12, 24, and 48 h were 3.6%, 1.9%, 3.1%, 1.0%, and 0.4%, respectively, whereas the corresponding values in the salt-treatment group were 16.9%, 15.1%, 27.5%, 11.9%, and 11.1%. The highest •OH radical-scavenging activity of IPS was observed at 12 h. Similarly, the •OH radical-scavenging activity of EPS was also higher in the salt-treatment group than in the control group throughout the treatment period, with a maximum value of 7.5% (Figure 3d).

The DPPH radical-scavenging activity of IPS was also higher in the salt-treatment group than in the control group throughout the treatment period (Figure 3e). In the control group, the values at 3, 6, 12, 24, and 48 h were 10.3%, 15.6%, 12.0%, 11.5%, and 17.1%, respectively, whereas those in the salt-treatment group were 35.8%, 34.3%, 33.6%, 31.2%, and 34.3%. The DPPH radical-scavenging activity of EPS showed the same pattern. The values in the control group ranged from 3.6% to 9.2%, whereas those in the salt-treatment group ranged from 23.5% to 24.9% (Figure 3f).

In contrast, the reducing power of IPS was lower in the salt-treatment group than in the control group at all time points after stress treatment began (Figure 3g). At 0 h, the absorbance at 700 nm was similar in the two groups. In the control group, the values at 3, 6, 12, 24, and 48 h were 0.14, 0.15, 0.14, 0.15, and 0.16, respectively, whereas the corresponding values in the salt-treatment group were 0.10, 0.08, 0.08, 0.07, and 0.08. The reducing power of IPS in the salt-treatment group showed an overall decline with prolonged stress duration. The reducing power of EPS showed only minor fluctuations over time, and the difference between the control and salt-treatment groups varied across time points (Figure 3h).

### 3.5. Temporal Transcriptomic Variation in M. sextelata Strain Mel-21 During NaCl Exposure

#### 3.5.1. RNA-Seq Data Overview and Differential Gene Expression Analysis

RNA-seq analysis was performed to characterize temporal transcriptional variation in *M. sextelata* Mel-21 during exposure to 300 mmol·L^−1^ NaCl relative to the common 0 h baseline. All libraries showed high sequencing quality, with Q20 values of 97.65–98.04%, Q30 values of 92.96–93.93%, and clean-base retention rates of 94.52–95.42%. Total alignment rates ranged from 98.19% to 98.72%, and unique alignment rates ranged from 97.35% to 98.01%. These metrics describe read quality; they do not address the absence of time-matched untreated controls.

**Figure 4:**
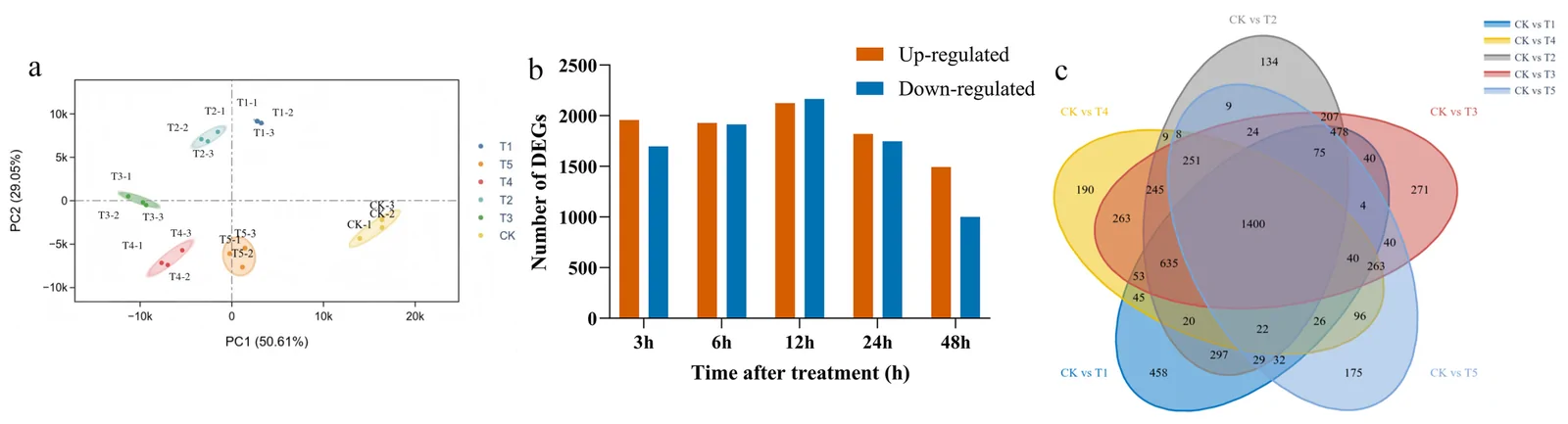
Temporal transcriptomic variation in *M. sextelata* Mel-21 during exposure to 300 mmol·L^−1^ NaCl. (**a**) Principal component analysis of the RNA-seq samples; (**b**) numbers of up- and downregulated differentially expressed genes (DEGs) in the 3 h versus 0 h, 6 h versus 0 h, 12 h versus 0 h, 24 h versus 0 h, and 48 h versus 0 h comparisons; and (**c**) Venn diagram showing DEGs shared among the five comparisons.

PCA revealed distinct clustering patterns between the initial 0 h baseline and the samples collected at different times during NaCl exposure (Figure 4a). The 3 h and 6 h samples clustered closely, whereas the 12 h samples showed marked differentiation from both early and later treatment stages. The 24 h and 48 h samples were positioned relatively close to each other, indicating partial similarity during prolonged salt exposure. These results indicate that Mel-21 exhibited distinct time-dependent transcriptional changes during NaCl exposure relative to the initial 0 h baseline.

The number of DEGs varied across treatment time points. A total of 3654, 3843, 4289, 3566, and 2494 DEGs were identified at 3, 6, 12, 24, and 48 h, respectively (Figure 4b). The largest number of DEGs occurred at 12 h, indicating that this time point represented a pronounced transcriptional response stage under the present experimental conditions. Venn analysis identified 1400 DEGs shared by all five treatment time points (Figure 4c), suggesting the presence of a core set of genes that were differentially expressed during NaCl exposure relative to the initial 0 h baseline.

#### 3.5.2. GO Functional Enrichment Analysis of DEGs

**Figure 5:**
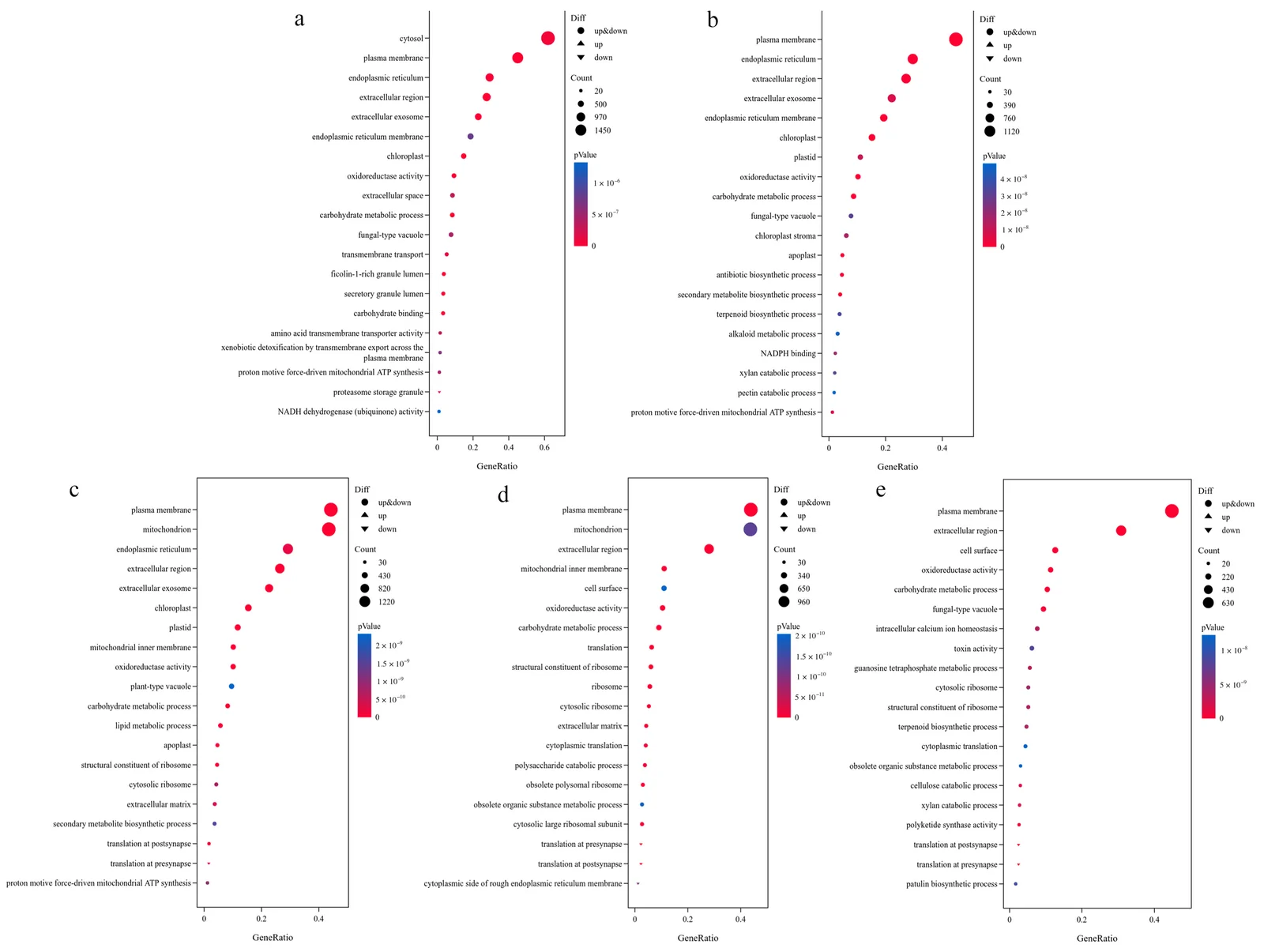
GO enrichment analysis of genes differentially expressed during NaCl exposure relative to the common 0 h baseline in *M. sextelata* Mel-21. (**a**–**e**) Comparisons of 3, 6, 12, 24, and 48 h with the 0 h baseline, respectively. DEGs were defined by |log_2_(fold change)| ≥ 1 and adjusted *p* value < 0.05. The horizontal axis shows GeneRatio, point size indicates the number of DEGs assigned to each GO term, and color represents the nominal enrichment *p* value. Upward triangles, downward triangles, and circles (“up&down”) indicate GO terms associated with upregulated DEGs only, downregulated DEGs only, or both, respectively.

GO enrichment analysis summarized functional categories represented among DEGs identified during NaCl exposure relative to the common 0 h baseline; the plot symbols distinguish terms associated with upregulated genes, downregulated genes, or both (Figure 5). Across the five treatment time points, enriched GO terms were mainly associated with membrane-associated components, extracellular region, oxidoreductase activity, carbohydrate metabolism, ribosome-related processes, calcium ion homeostasis, and cell-wall-related remodeling. These results identify functional categories represented among genes differentially expressed during NaCl exposure relative to the common 0 h baseline.

At early treatment stages, enriched terms were mainly related to membrane transport, cytosolic and endoplasmic-reticulum-associated components, and carbohydrate-related processes. At 12 h, terms associated with mitochondrial function, lipid metabolism, and ribosome structure became more prominent. At later stages, DEGs were enriched in terms related to translation, vacuole-associated regulation, calcium ion homeostasis, and cell wall carbohydrate degradation.

#### 3.5.3. KEGG Pathway Enrichment Analysis of DEGs

**Figure 6:**
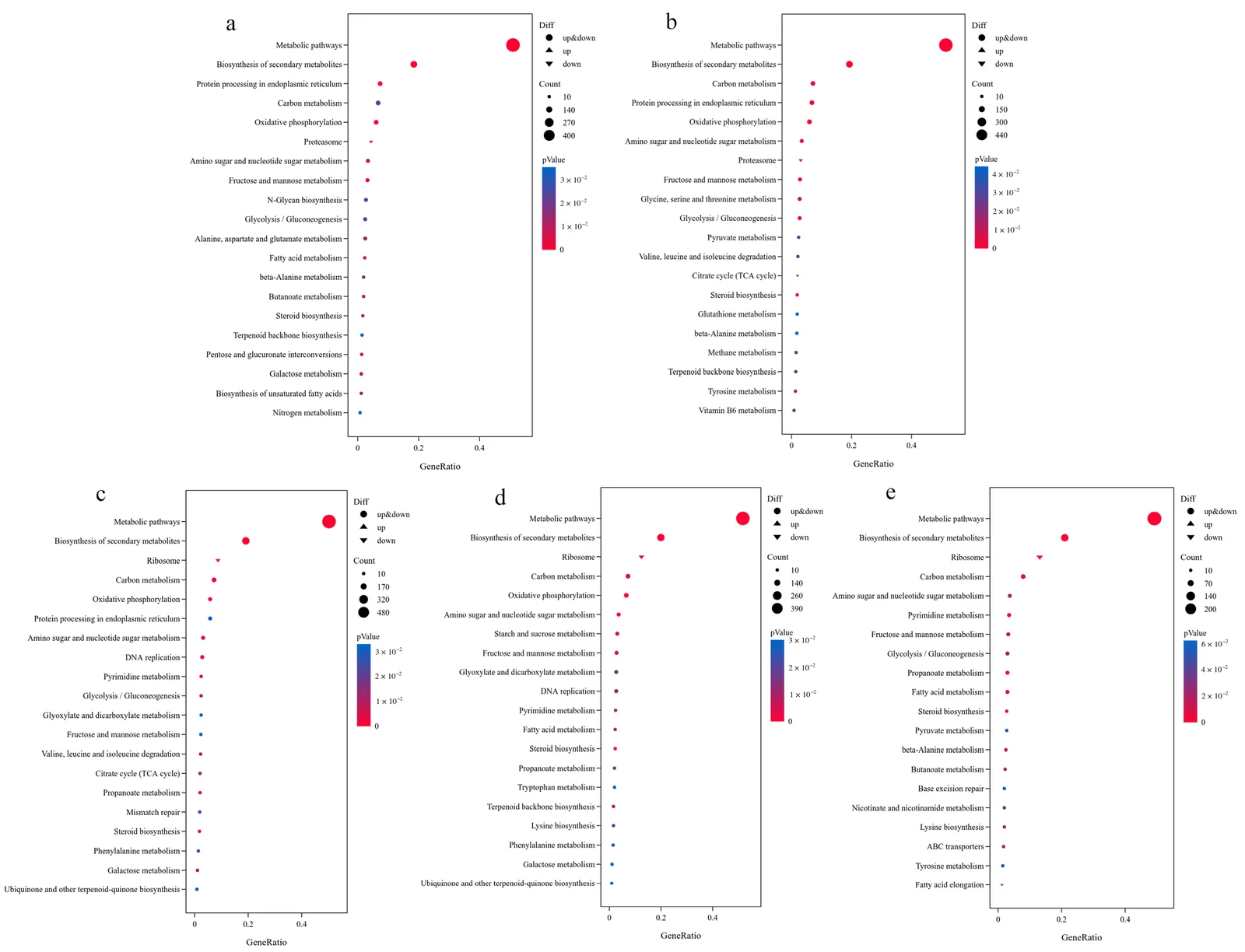
KEGG enrichment analysis of genes differentially expressed during NaCl exposure relative to the common 0 h baseline in *M. sextelata* Mel-21. (**a**–**e**) Comparisons of 3, 6, 12, 24, and 48 h with the 0 h baseline, respectively. The horizontal axis shows GeneRatio, point size indicates the number of DEGs assigned to each pathway, and color indicates the nominal enrichment *p* value. Upward triangles, downward triangles, and circles (“up&down”) indicate pathways associated with upregulated DEGs only, downregulated DEGs only, or both, respectively.

KEGG analysis showed that DEGs identified relative to the common 0 h baseline were enriched in pathways related to primary metabolism, carbon metabolism, amino sugar and nucleotide sugar metabolism, fructose and mannose metabolism, oxidative phosphorylation, protein processing in the endoplasmic reticulum, ribosome, lipid metabolism, nucleotide metabolism, DNA repair, and ABC transporters (Figure 6). These enrichment patterns identify functional categories represented among DEGs relative to the 0 h baseline, but do not directly demonstrate pathway activation or increased metabolic flux.

At the early sampling points, enriched pathways were mainly related to protein processing, oxidative phosphorylation, glycolysis/gluconeogenesis, fatty acid metabolism, and nitrogen metabolism. At 12 h, pathways related to ribosome, nucleotide metabolism, oxidative phosphorylation, and protein processing were strongly represented, consistent with the high DEG number at this stage. At later sampling points, DEGs were enriched in pathways related to base excision repair, ABC transporters, fatty acid metabolism, and carbohydrate metabolism.

#### 3.5.4. Candidate Genes Identified During NaCl Exposure

Based on functional annotation and expression patterns relative to the common 0 h baseline, 25 DEGs were selected as candidates for further investigation in *M. sextelata* (Table 4). These genes were mainly associated with ion transport, osmotic regulation, ROS scavenging, protein folding and quality control, ubiquitin-mediated protein turnover, autophagy, signal transduction, transcriptional regulation, and energy metabolism. Most candidate DEGs showed consistent up- or downregulation across the five comparisons with the common 0 h baseline. The annotation information, expression patterns, and functional categories of these 25 candidate DEGs are summarized in Table 4.

#### 3.5.5. qRT-PCR Validation of RNA-Seq Results

To assess agreement with the RNA-seq expression patterns, 12 DEGs were examined using qRT-PCR with the same common 0 h reference (Figure S9). The qRT-PCR results showed expression trends consistent with those obtained from RNA-seq analysis, with a strong positive correlation (*R*^2^ = 0.93).

#### 3.5.6. Weighted Gene Co-Expression Network Analysis

WGCNA was performed using 18 RNA-seq libraries with a soft-thresholding power of 24, the lowest tested value achieving an approximately scale-free topology fit index above 0.85. The module–trait relationship heatmap is presented in Figure S10. The two culture sets were matched using the original experimental pairing records. Because the physiological measurements and RNA-seq data originated from different members of each culture pair, the displayed relationships were interpreted as exploratory associations within the time-course dataset. The lightcyan, brown, darkred, grey60, and darkslateblue modules were selected because of their relatively high absolute module–trait correlations, without BH correction of the correlation tests. Their KEGG enrichment results are presented in Figure S11 to summarize their functional composition.

The five modules showed distinct functional enrichment patterns. Genes in the darkred module were mainly enriched in membrane transport, vesicular trafficking, stress signaling, and protein turnover pathways. The grey60 module showed enrichment in ribosome, autophagy, SNARE interactions in vesicular transport, and endocytosis pathways. The brown module was mainly enriched in oxidative phosphorylation, inositol phosphate metabolism, and other metabolic pathways. The lightcyan module showed enrichment in ribosome biogenesis, ABC transporters, fatty acid degradation, and amino acid metabolism. The darkslateblue module was enriched in autophagy, ubiquitin-mediated proteolysis, and endocytosis pathways. These results describe the functional composition of the examined modules within the NaCl-exposure time-course dataset and do not establish regulatory effects on the physiological traits.

### 3.6. Heterologous Expression Analysis of Gene05935

Candidate genes were screened according to predefined criteria, including consistent upregulation relative to the common 0 h baseline at all five exposure time points, an FPKM mean ≥ 3 at each stage, log_2_FC ≥ 3.5 at each stage, mean log_2_FC ≥ 5, and functional annotation associated with salt stress response, transport, or stress-response-related processes. Based on these criteria, *Gene05935* was selected as a priority candidate gene.

#### 3.6.1. Physicochemical Properties and Functional Prediction of Proteins

Sequence analysis revealed that *Gene05935* encodes a 414-amino-acid protein with a predicted molecular weight of 44.79 kDa. Hydrophobicity and transmembrane topology analyses identified ten putative transmembrane helices, consistent with a membrane-associated protein (Figure S12). These predictions support the description of *Gene05935* as a candidate multi-pass transmembrane protein. However, these bioinformatic predictions do not demonstrate ion-channel activity or identify its transported substrate.

#### 3.6.2. Preliminary Qualitative Observations of Transgenic *A. thaliana* Seedlings

T3 transgenic *A. thaliana* plants carrying the *Gene05935* construct were examined under NaCl treatment, with empty-vector plants used as controls. *Gene05935* amplification occurred earlier in transgenic samples than in empty-vector controls (Figure S13a). Conventional PCR followed by agarose gel electrophoresis showed no visible amplification band in the empty-vector controls. In the qRT-PCR assay, these controls exhibited late amplification near Cq 39, which may represent nonspecific amplification.

**Figure 7:**
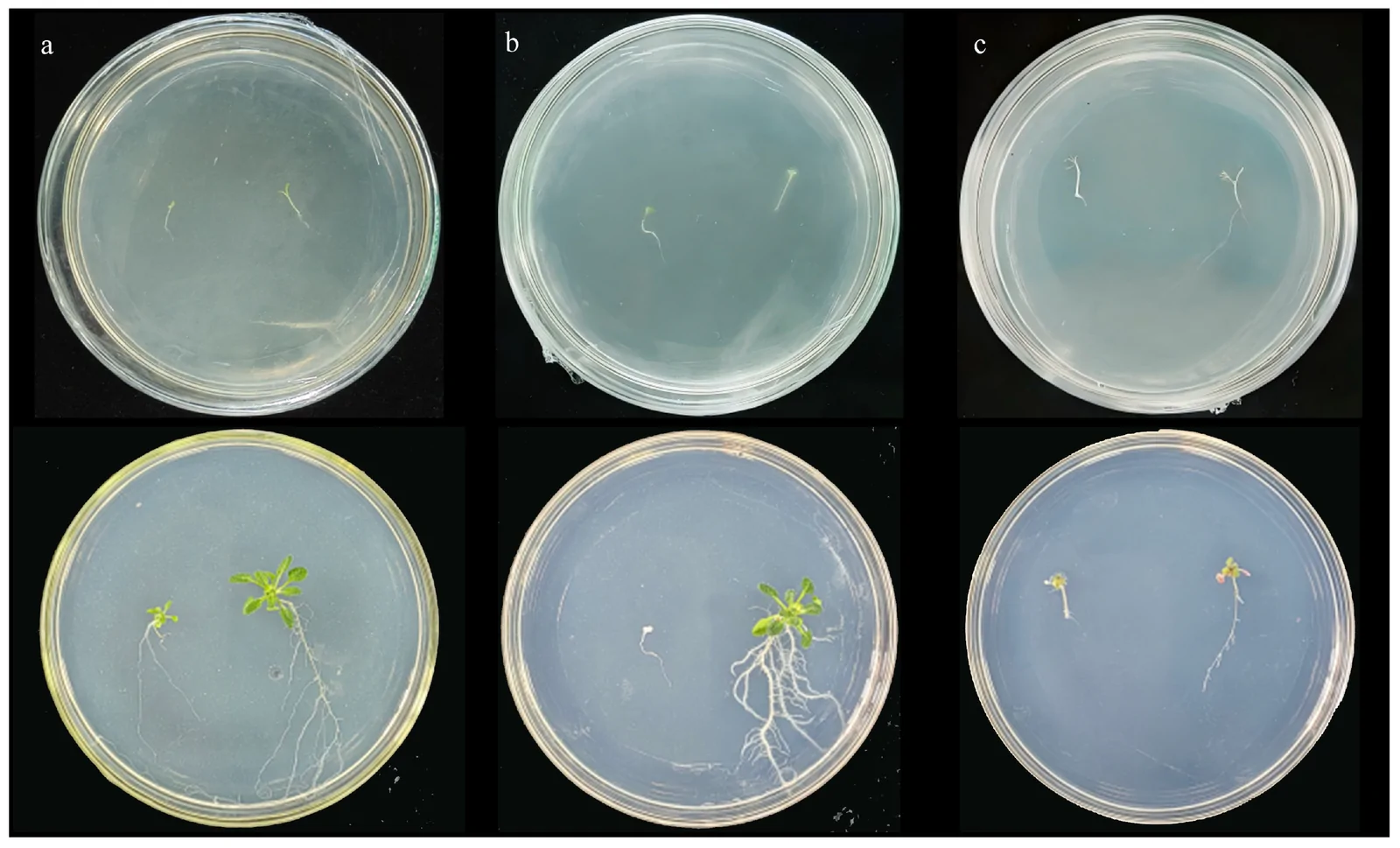
Representative photographs of transgenic *Arabidopsis thaliana* seedlings carrying the *Gene05935* construct and empty-vector controls before and after NaCl exposure. (**a**) 100 mmol·L^−1^ NaCl; (**b**) 200 mmol·L^−1^ NaCl; and (**c**) 300 mmol·L^−1^ NaCl. Each panel shows one representative empty-vector control seedling on the left and one representative seedling carrying the *Gene05935* construct on the right. The upper and lower rows show seedlings before treatment and after 15 days of NaCl exposure, respectively. Three replicate sets each included all three NaCl concentrations (three plates per concentration; nine plates and 18 seedlings in total), with exactly two seedlings on each plate: one empty-vector control and one *Gene05935*-transgenic seedling. Photographs were selected following non-blinded visual assessment and provide a qualitative illustration rather than a quantitative comparison of salt tolerance.

Representative photographs taken before treatment and after 15 days of NaCl exposure illustrate the appearance of seedlings carrying the *Gene05935* construct and empty-vector controls (Figure 7). Visual differences were observed between the photographed seedlings, but the assessment was non-blinded and no quantitative phenotypic endpoints were measured. These images are therefore presented as preliminary qualitative observations and do not establish a reproducible enhancement of salt tolerance.

#### 3.6.3. Heterologous Expression of *Gene05935* in *S. cerevisiae*

To examine growth associated with heterologous *Gene05935* expression under NaCl exposure, *Gene05935* was heterologously expressed in *S. cerevisiae* using the pYES2 vector, with empty-vector strains used as controls. qRT-PCR amplification signals were detected earlier in the *Gene05935*-expressing yeast samples than in the empty-vector controls (Figure S13b). Conventional PCR followed by agarose gel electrophoresis showed no visible amplification band in the empty-vector controls. In the qRT-PCR assay, these controls exhibited late amplification near Cq 39, which may represent nonspecific amplification. At 2.0 and 2.5 mol·L^−1^ NaCl, the *Gene05935*-expressing strain showed higher mean endpoint OD_600_ values than the empty-vector control after 24 h of incubation (Figure S14). The liquid assay included higher NaCl concentrations than the spot assay, so the two assays do not provide a direct comparison at 2.0 and 2.5 mol·L^−1^ NaCl.

**Figure 8:**
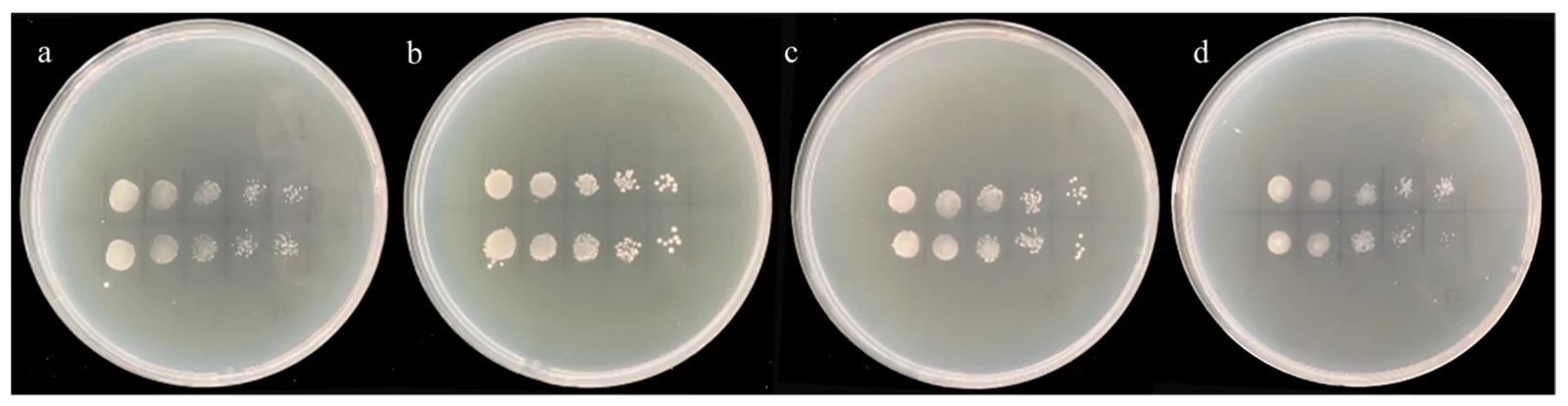
Growth performance of *Saccharomyces cerevisiae* expressing *Gene05935* under NaCl stress. Yeast strains harboring pYES2-*Gene05935* (upper rows) or the empty pYES2 vector (lower rows) were serially diluted and spotted onto selective agar plates supplemented with (**a**) 0 mol·L^−1^, (**b**) 0.5 mol·L^−1^, (**c**) 1.0 mol·L^−1^, or (**d**) 1.5 mol·L^−1^ NaCl. In each panel, spots from left to right represent serial tenfold dilutions of 10^0^, 10^−1^, 10^−2^, 10^−3^, and 10^−4^, respectively. Cultures were adjusted to OD_600_ = 1.0 before dilution, and 5 μL of each dilution was spotted. Plates were incubated at 30 °C for 5 days before imaging. The assay was independently repeated three times.

Salt tolerance was assessed by spot dilution assays on NaCl-supplemented media. As shown in Figure 8, the *Gene05935*-expressing strain and the empty-vector control exhibited similar growth under 0 mol·L^−1^ NaCl. Under increasing NaCl concentrations, the empty-vector strain displayed stronger growth inhibition, whereas the *Gene05935*-expressing strain maintained visibly better growth performance (Figure 8). These results indicate that heterologous expression of *Gene05935* was associated with improved yeast growth performance under NaCl stress.

## 4. Discussion

### 4.1. Integrated Evaluation Identifies Mel-21 and a Representative Salt-Stress Condition

Salt tolerance in filamentous fungi is a complex quantitative trait determined by coordinated changes in growth, osmotic adjustment, membrane stability, ion homeostasis, redox regulation, and metabolic activity. Evaluation based on a single indicator may therefore provide an incomplete estimate of the overall stress-tolerance capacity of a strain. In the present study, correlation analysis, principal component analysis (PCA), and membership function analysis were combined to integrate multiple growth-related traits. This approach summarized the selected traits into a composite measure of growth-related performance under the screening conditions; its interpretation depends on the selected variables and their weighting [21,22].

Among the 24 preselected isolates representing four *Morchella* species, *M. sextelata* Mel-21 obtained the highest composite screening score under 1 mol·L^−1^ NaCl, supporting its selection for subsequent analyses. This ranking was reconstructed from the rounded coefficients reported in Table S7, includes interspecific variation, and is specific to the tested isolates, selected traits, and screening conditions. Closely spaced scores should be interpreted cautiously.

Selection of an appropriate stress intensity is critical because mild treatments may induce only limited responses, whereas excessively severe treatments may obscure adaptive regulation. The selection of 300 mmol·L^−1^ NaCl was primarily based on the physiological observations: mycelial growth rate decreased by 51.32% relative to the control, while measurable growth was maintained and clear physiological responses were observed. This balance between pronounced stress responses and continued growth supported its use as a working concentration for subsequent time-course experiments. PCA provided a supplementary overview of how growth-related and other physiological traits varied across NaCl concentrations. However, growth inhibition rate was derived from mycelial growth rate, and including both variables gives additional weight to growth-related variation. The PCA was therefore interpreted descriptively rather than as independent confirmation of trait relationships or of the selected stress concentration.

### 4.2. Salt Stress Imposes Coupled Osmotic, Ionic, Membrane, and Oxidative Constraints

NaCl treatment enabled assessment of the integrated mycelial response to salinity, encompassing both osmotic and ionic effects. This integrated response reflects the multiple physiological constraints imposed by saline conditions. High external salinity lowers water availability and disrupts cellular turgor, thereby restricting polarized hyphal extension. Because filamentous fungal growth depends on coordinated membrane expansion, vesicular transport, cytoskeletal organization, and cell-wall synthesis, disturbance of these processes can directly reduce colony expansion and biomass accumulation [37,38,39].

The progressive decrease in hyphal diameter further indicates that NaCl stress affected structural development rather than only radial colony growth. Narrower hyphae may reflect reduced turgor-driven expansion, altered cell-wall deposition, or limitations in membrane and cytoplasmic biosynthesis. However, the absence of severe twisting, swelling, or fragmentation under 300 mmol·L^−1^ NaCl suggests that Mel-21 retained basic hyphal integrity during the time-course experiment. Thus, the observed morphological response appears to represent constrained growth and structural adjustment.

Increased electrical conductivity provides additional evidence that NaCl stress impaired membrane integrity. The sharp increase between 200 and 300 mmol·L^−1^ NaCl suggests that membrane permeability became substantially altered within this concentration range. Increased permeability may disrupt nutrient uptake, membrane potential, ion compartmentalization, and membrane-bound enzymatic reactions. Malondialdehyde (MDA) content increased under moderate salt stress and subsequently declined at higher concentrations, indicating a non-linear response of membrane lipid peroxidation to increasing salinity.

Proline and soluble proteins showed concentration-dependent accumulation patterns, indicating that osmotic adjustment contributed to the response of Mel-21. Proline can function as a compatible solute and may also contribute to membrane stabilization, protein protection, and redox regulation [40]. Its maximum accumulation at 300 mmol·L^−1^ NaCl may reflect a marked physiological response to NaCl exposure. Soluble protein accumulation under moderate salinity may similarly reflect the synthesis or retention of stress-related proteins, whereas the decline under more severe stress may be associated with changes in protein synthesis, degradation, and overall metabolic activity.

Ion imbalance was another prominent feature of the response. Increasing Na^+^ accumulation, accompanied by marked decreases in the K^+^/Na^+^ and Ca^2+^/Na^+^ ratios, indicates that NaCl exposure disrupted intracellular ion homeostasis. Maintenance of a favorable K^+^/Na^+^ ratio is important for enzyme activity, membrane potential, protein synthesis, and primary metabolism [41]. Excess Na^+^ may compete with K^+^ uptake or interfere with K^+^-dependent cellular processes. The reduction in the Ca^2+^/Na^+^ ratio may also affect calcium-dependent signaling and membrane stabilization.

The responses of superoxide dismutase, peroxidase, and catalase further indicate that antioxidant enzyme activities changed under NaCl stress. Their activities generally increased under low to moderate salinity and declined under more severe stress. This non-linear pattern suggests that antioxidant capacity varied with both stress intensity and cellular metabolic status. Taken together, these results indicate that the physiological response of Mel-21 involved concurrent changes in osmotic adjustment, membrane stability, ion homeostasis, and antioxidant enzyme activities.

### 4.3. Temporal Patterns of Polysaccharide Content and Crude-Extract Antioxidant Activity Under Salt Stress

Fungal polysaccharides contribute to carbon storage, osmotic regulation, extracellular protection, and antioxidant activity. In the present study, intracellular polysaccharides (IPS) and extracellular polysaccharides (EPS) showed distinct concentration- and time-dependent responses, indicating that intracellular and extracellular polysaccharides participate differently in the response of *M. sextelata* to salt stress.

Under the NaCl concentration gradient, IPS content decreased progressively, whereas EPS content increased under low to moderate salinity and subsequently declined at higher concentrations. These contrasting patterns indicate that salt stress altered the allocation and accumulation of intracellular and extracellular carbohydrate components. During the 300 mmol·L^−1^ NaCl time-course experiment, IPS showed rapid early accumulation followed by a gradual decline, whereas EPS exhibited a different temporal response pattern.

The crude IPS and EPS extracts showed increased radical-scavenging activities under NaCl treatment even when polysaccharide contents did not increase. These results describe antioxidant activity of polysaccharide-rich extracts. Co-extracted constituents may also contribute to the measured activities. Possible relationships between polysaccharide structural characteristics and antioxidant activity have been discussed in previous studies [14,42,43], but were not tested in the present study.

### 4.4. Time-Dependent Transcriptional Variation During NaCl Exposure

The PCA pattern suggests similarities in transcriptional profiles between 3 and 6 h and between 24 and 48 h. The separation of the 12 h samples, together with the largest number of DEGs relative to the 0 h baseline, suggests a distinct transcriptional state at this sampling point.

The enrichment results indicate that transcriptional differences relative to the common 0 h baseline involved multiple functional categories during NaCl exposure. At early sampling points, enriched categories included protein processing, oxidative phosphorylation, glycolysis/gluconeogenesis, fatty-acid metabolism, nitrogen metabolism, and membrane-associated processes. These categories suggest possible links with energy metabolism and biosynthetic processes that warrant further investigation. Maintenance of protein folding and endoplasmic-reticulum function may be particularly important because salt-induced ionic and oxidative stress can impair protein stability.

At 12 h, DEGs were prominently represented in ribosome-related processes, nucleotide metabolism, oxidative phosphorylation, and protein processing. At later sampling points, enriched categories included DNA repair, ABC transporters, fatty-acid metabolism, autophagy, and vesicular transport. Previous studies have linked these processes to cellular maintenance and stress responses [44,45,46,47,48], providing possible biological context for the observed expression patterns.

WGCNA provided a descriptive overview of co-expression patterns during NaCl exposure [16,34]. Functional enrichment of the five examined modules highlighted membrane transport, vesicular trafficking, ribosome function, autophagy, endocytosis, oxidative phosphorylation, fatty-acid degradation, and ubiquitin-mediated proteolysis. Physiological measurements and RNA sequencing were conducted using distinct cultures paired at the start of the experiment and matched according to the original pairing records. The module–trait relationships may reflect shared temporal patterns and should therefore be interpreted as exploratory associations rather than evidence of direct regulation or independent functional validation. In addition, the absence of time-matched untreated RNA-seq controls prevents separation of NaCl-specific transcriptional effects from cultivation-related temporal changes; enrichment patterns therefore identify processes for further investigation rather than established mechanisms of salt adaptation.

### 4.5. Gene05935 Is a Candidate Transmembrane Protein Identified During NaCl Exposure

*Gene05935* was selected because its transcript abundance was consistently higher at all five NaCl-exposure time points than at the common 0 h baseline and it encoded a predicted hydrophobic protein with ten transmembrane helices. Its predicted multi-pass transmembrane topology supports further investigation of its potential membrane-associated role.

In plants, SLAC1 and SLAH proteins function as anion channels involved in membrane-potential regulation, anion transport, stomatal movement, and abiotic stress responses [49,50]. These studies provide a comparative context for investigating membrane proteins associated with stress responses. Assignment of *Gene05935* to the SLAC1/SLAH family requires additional sequence-based evidence.

In yeast, *Gene05935* expression was associated with higher mean endpoint OD_600_ values at 2.0 and 2.5 mol·L^−1^ NaCl. The 24 h OD_600_ measurements describe endpoint culture turbidity and do not distinguish differences in lag phase, growth rate, or final biomass. The *Arabidopsis* assay provided supplementary qualitative observations of seedling appearance under NaCl exposure. Because the plant assessment was non-blinded and lacked quantitative phenotypic measurements and a separately reported comparison among independent transgenic events, it does not establish enhanced salt tolerance in *Arabidopsis*. Quantitative evaluation of independently derived transgenic plants would be needed to determine whether the observed visual differences are reproducible. Furthermore, the predicted transmembrane structure and the yeast growth observations are insufficient to demonstrate direct ion transport or ion-channel activity. Subcellular localization, electrophysiological measurements, ion-flux assays, and substrate-specific transport experiments are required to determine the protein’s membrane localization and transport function.

## 5. Conclusions

Among the 24 isolates preselected from the initial collection of 48 isolates, *M. sextelata* Mel-21 obtained the highest comprehensive screening score. Salt stress inhibited mycelial growth and induced coordinated changes in membrane integrity, osmotic adjustment, ion homeostasis, polysaccharide metabolism, and antioxidant defense. Integrated physiological analysis identified 300 mmol·L^−1^ NaCl as a representative stress condition that induced pronounced physiological responses while retaining measurable mycelial growth. Time-course analysis showed changes in polysaccharide accumulation and antioxidant activities, while RNA-seq identified the largest number of DEGs relative to the common 0 h baseline at 12 h. Relative to the common 0 h baseline, the transcriptomic analyses identified temporal expression differences involving membrane-related processes, carbohydrate and energy metabolism, oxidoreductase activity, protein processing, vesicular transport, autophagy, and transport-related pathways. These patterns cannot be attributed specifically to NaCl because time-matched untreated controls were not included. The physiological measurements and temporal transcriptional profiles provide a basis for further investigation of *M. sextelata* under saline conditions. *Gene05935* remains a candidate for further study, supported by the higher mean endpoint OD_600_ values observed in yeast; the *Arabidopsis* photographs provide preliminary qualitative observations rather than functional validation of enhanced salt tolerance. These results may also inform future strain-improvement programs and protected-cultivation trials using selected *Morchella* inocula under mildly saline or marginal-soil conditions.

## Figures and Tables

**Table 1:** Reconstructed composite screening scores of 24 *Morchella* strains using PCA-informed grouping and membership-function analysis.

Serial Number	Comprehensive Index		Subordinative Function		*D* Value	Salt-Tolerance Rank
	CI_1_	CI_2_	X_1_	X_2_		
1	0.79	1.60	0.2842	0.7512	0.37939	12
2	0.60	1.80	0.2158	0.8451	0.34413	19
7	0.90	1.58	0.3237	0.7418	0.40898	9
8	0.72	1.55	0.2590	0.7277	0.35456	17
9	0.63	1.25	0.2266	0.5869	0.30007	22
10	1.36	1.20	0.4892	0.5634	0.50433	7
11	0.61	1.57	0.2194	0.7371	0.32498	21
12	1.70	1.60	0.6115	0.7512	0.63999	5
13	0.85	1.16	0.3058	0.5446	0.35446	18
14	0.70	1.40	0.2518	0.6573	0.33448	20
15	1.35	1.59	0.4856	0.7465	0.53880	6
21	2.78	1.60	1.0000	0.7512	0.94926	1
22	0.85	1.57	0.3058	0.7371	0.39370	10
23	0.73	1.80	0.2626	0.8451	0.38136	11
27	1.56	2.04	0.5612	0.9577	0.64202	4
29	0.63	1.96	0.2266	0.9202	0.36804	15
31	0.86	1.97	0.3094	0.9249	0.43486	8
35	0.00	0.00	0.0000	0.0000	0.00000	23
36	0.79	1.35	0.2842	0.6338	0.35546	16
38	2.35	2.04	0.8453	0.9577	0.86825	2
39	0.77	1.60	0.2770	0.7512	0.37367	13
40	1.99	2.13	0.7158	1.0000	0.77377	3
45	0.00	0.00	0.0000	0.0000	0.00000	23
46	0.85	1.35	0.3058	0.6338	0.37264	14
Weight			79.61%	20.39%		

Note: Calculations are based on the rounded coefficients reported in Table S7. CI1 = MD + SN + FW + DW; CI2 = GR + FWC + GT. Each index was min–max-normalized across the 24 isolates, and *D* = 0.7961X_1_ + 0.2039X_2_. Ranks were calculated before rounding the displayed scores; tied scores share the same rank. Reconstruction from rounded inputs can affect closely spaced ranks.

**Table 2:** Effects of different NaCl concentrations on mycelial biomass of *M. sextelata*.

NaCl Concentration (mmol·L^−1^)	Fresh Weight (g)	Dry Weight (g)	Water Content (%)
0	27.56 ± 1.50 ^a^	1.21 ± 0.02 ^a^	95.62 ± 0.16 ^a^
100	19.23 ± 0.36 ^b^	0.81 ± 0.01 ^b^	95.78 ± 0.13 ^a^
200	7.72 ± 0.78 ^c^	0.32 ± 0.02 ^c^	95.79 ± 0.41 ^a^
300	1.56 ± 0.41 ^d^	0.12 ± 0.04 ^d^	94.25 ± 0.70 ^a b^
400	1.47 ± 0.51 ^d^	0.09 ± 0.01 ^d^	91.73 ± 0.14 ^b^
500	0.82 ± 0.03 ^d^	0.07 ± 0.01 ^d^	91.58 ± 0.22 ^b^

Note: Values are means ± SD (*n* = 3 independent PDA plates). After a 2-day preculture, all treatments were initiated simultaneously and harvested when the control colonies covered the plates. Different lowercase letters within a column indicate significant differences among NaCl concentrations (*p* < 0.05; one-way ANOVA followed by Tukey’s test).

**Table 3:** PCA of the listed physiological indices of *M. sextelata* mycelia under NaCl stress, excluding EPS.

Index	Component 1	Component 2	Component 3
Mycelial growth rate	0.988	−0.046	0.124
Inhibition ratio	−0.988	0.046	−0.124
Mycelial diameter	0.985	−0.031	0.108
Osmotic pressure	−0.974	−0.002	−0.211
Electrical conductivity	−0.997	0.037	0.049
Dry weight	0.927	−0.366	0.012
Fresh weight	0.936	−0.339	−0.021
MDA content	0.652	0.661	−0.37
Proline content	−0.823	0.445	0.326
IPS content	0.876	−0.441	0.13
Soluble protein content	0.066	0.705	0.577
SOD activity	0.469	0.628	0.450
POD activity	0.434	0.803	−0.392
CAT activity	0.625	0.713	−0.291
Eigenvalue	9.273	3.129	1.124
Contribution rate (%)	66.237	22.347	8.027

Note: The input comprised 18 biological-replicate observations (six concentrations × three replicates); variables were Z-score standardized before PCA. Abbreviations: PC, principal component; MDA, malondialdehyde; IPS, intracellular polysaccharide; SOD, superoxide dismutase; POD, peroxidase; CAT, catalase. Growth inhibition rate was derived from mycelial growth rate; their opposite loadings reflect this mathematical dependence. Both variables were retained for descriptive presentation, with the resulting additional weight on growth-related variation acknowledged in Section 4.1. This analysis excludes EPS and is separate from the EPS-inclusive PCA shown in Figure S5; the explained-variance percentages apply only to the analysis reported here.

**Table 4:** Functional annotations and expression changes of 25 differentially expressed genes consistently identified in five comparisons with the common 0 h baseline during NaCl exposure in *M. sextelata* Mel-21.

Gene ID	Functional Annotation	log_2_FC Relative to 0 h				
		3 h	6 h	12 h	24 h	48 h
*Gene02540*	P-type ATPase	7.42	7.16	6.55	7.63	2.95
*Gene03005*	ATP synthase delta	−1.41	−1.69	−2.01	−1.71	−1.03
*Gene03098*	Pyruvate dehydrogenase E1 alpha	−1.58	−1.34	−1.25	−1.28	−1.05
*Gene03282*	Glutathione peroxidase	1.46	1.25	1.80	1.69	1.61
*Gene03558*	DnaJ chaperone	3.67	3.81	3.42	2.48	2.68
*Gene03815*	Na^+^/H^+^ antiporter	3.92	2.86	1.65	1.11	1.19
*Gene04154*	E3 ubiquitin ligase	1.45	2.07	1.92	1.52	1.50
*Gene04534*	Glutathione S-transferase	5.44	3.97	2.91	2.19	2.69
*Gene04718*	bZIP transcription factor	1.89	2.09	2.25	2.24	1.25
*Gene04723*	Myo-inositol transporter	1.59	2.12	3.12	3.14	1.07
*Gene04963*	Ubiquitin-conjugating enzyme E2	2.61	3.43	3.75	3.10	2.25
*Gene05573*	Polyubiquitin	2.38	2.60	2.66	2.42	1.90
*Gene05698*	ATG1 kinase	2.56	2.90	2.75	2.69	2.00
*Gene05801*	Mn-superoxide dismutase	−1.03	−1.25	−1.92	−1.76	−1.03
*Gene05935*	Putative transmembrane protein	3.97	3.97	6.37	6.79	7.04
*Gene06512*	Catalase	4.89	4.45	5.27	4.77	4.37
*Gene07964*	Calcineurin subunit B	−1.57	−1.72	−1.83	−1.86	−1.05
*Gene09944*	Alternative oxidase	5.23	5.07	4.46	3.62	3.33
*Gene10621*	Aquaporin	−1.32	−1.47	−1.16	−1.33	−1.01
*Gene10664*	CorA-like Mg^2+^ transporter	1.46	2.03	2.57	3.02	1.89
*Gene10719*	DnaJ homolog	2.68	3.00	2.59	1.98	1.14
*Gene10722*	Ubiquitin-conjugating enzyme	7.68	7.72	7.47	6.70	5.86
*Gene11148*	Hsp70	−2.47	−3.14	−4.07	−4.24	−2.83
*Gene11818*	ATP synthase F_0_ subunit a	2.30	2.12	2.21	1.45	2.61
*MSTRG.15837.1*	NADH dehydrogenase subunit 2	3.12	2.24	1.37	1.98	3.48

Note: Abbreviation: log_2_FC, log_2_-transformed fold change relative to the 0 h baseline.

## Data Availability

The RNA-seq raw data generated in this study have been deposited in the NCBI Sequence Read Archive under BioProject accession number PRJNA1465190. Other data generated or analyzed during the current study are available from the corresponding author upon reasonable request.

## Supplementary Materials

The following supporting information can be downloaded at: https://www.mdpi.com/article/10.3390/jof12100736/s1, Figure S1, PCR amplification of *MAT1-1* and *MAT1-2* idiomorphs in 48 *Morchella* strains; Figure S2. Bayesian phylogenetic tree of the 24 selected *Morchella* isolates and reference sequences inferred from ITS sequences; Figure S3, mycelial growth of *M. sextelata* under different NaCl concentrations; Figure S4, hyphal morphology of *M. sextelata* under different NaCl concentrations; Figure S5, principal component analysis biplot of NaCl treatments and physiological traits in *M. sextelata* mycelia; Figure S6, time-dependent changes in hyphal morphology of *M. sextelata* Mel-21 under 300 mmol·L^−1^ NaCl treatment; Figure S7, time-dependent changes in hyphal diameter of *M. sextelata* Mel-21 under 300 mmol·L^−1^ NaCl treatment; Figure S8, time-dependent changes in the dry weight of *M. sextelata* Mel-21 mycelia in the control and 300 mmol·L^−1^ NaCl treatment groups; Figure S9, qRT-PCR validation of selected DEGs; Figure S10, module–trait relationships identified by weighted gene co-expression network analysis (WGCNA); Figure S11, KEGG pathway enrichment analysis of genes in five WGCNA modules selected for their relatively high absolute module–trait correlations; Figure S12, conserved-domain prediction of the protein encoded by *Gene05935*; Figure S13, qRT-PCR amplification curves for *Gene05935* in heterologous expression assays; Figure S14, Endpoint growth of *Saccharomyces cerevisiae* expressing *Gene05935* under different NaCl concentrations. Supplementary tables include: Table S1, original identifiers and species names recorded at collection for the 48 *Morchella* isolates used in this study; Table S2, primer sequences used for mating-type gene detection in *Morchella* strains; Table S3, evaluation of germination score under different treatments; Table S4, evaluation of mycelial density under different treatments; Table S5, evaluation of sclerotia density under different treatments; Table S6, primer sequences used for qRT-PCR; Table S7, salt stress response coefficients of physiological indices in 24 *Morchella* strains; Table S8, correlation matrix of salt tolerance coefficients for individual indices; Table S9, coefficients and contribution rates of the comprehensive index derived from PCA; Table S10, mycelial Na^+^ and K^+^ contents and K^+^/Na^+^ mass ratios in *M. sextelata* Mel-21 under different NaCl concentrations with 20 mmol·L^−1^ KCl supplementation; Table S11, mycelial Na^+^ and Ca^2+^ contents and Ca^2+^/Na^+^ mass ratios in *M. sextelata* Mel-21 under different NaCl concentrations with 20 mmol·L^−1^ CaCl_2_ supplementation.
