# Supplementary material for: Physiological Responses and Time-Course Transcriptomic Analysis of Morchella sextelata Strain Mel-21 During NaCl Exposure
Source: J Fungi (Basel). 2026 Oct 1;12(10):736. doi: 10.3390/jof12100736 (PMC13706175; doi:10.3390/jof12100736)
Supplement: Supplementary file 1 [file jof-12-00736-s001.zip › jof-4529282-supplementary.pdf]

Supplementary Figures

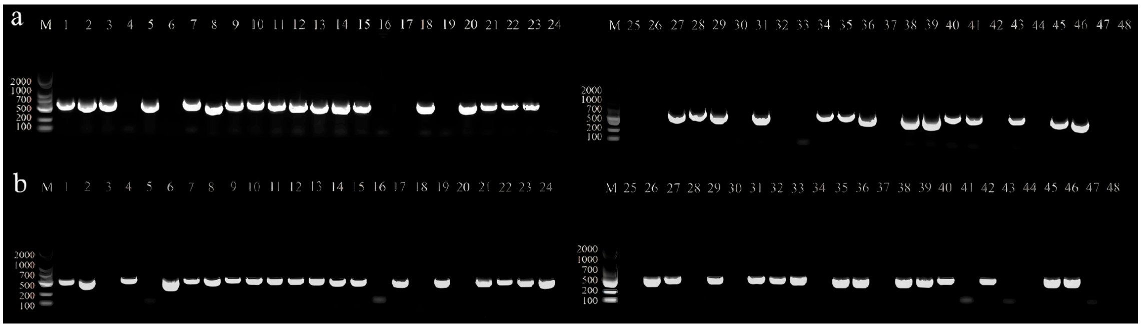

Figure S1. PCR amplification of *MAT1-1* and *MAT1-2* idiomorphs in 48 *Morchella* strains. Note: "M" is the standard Marker of DNA molecular weight, and "1-48" are the number of strains.

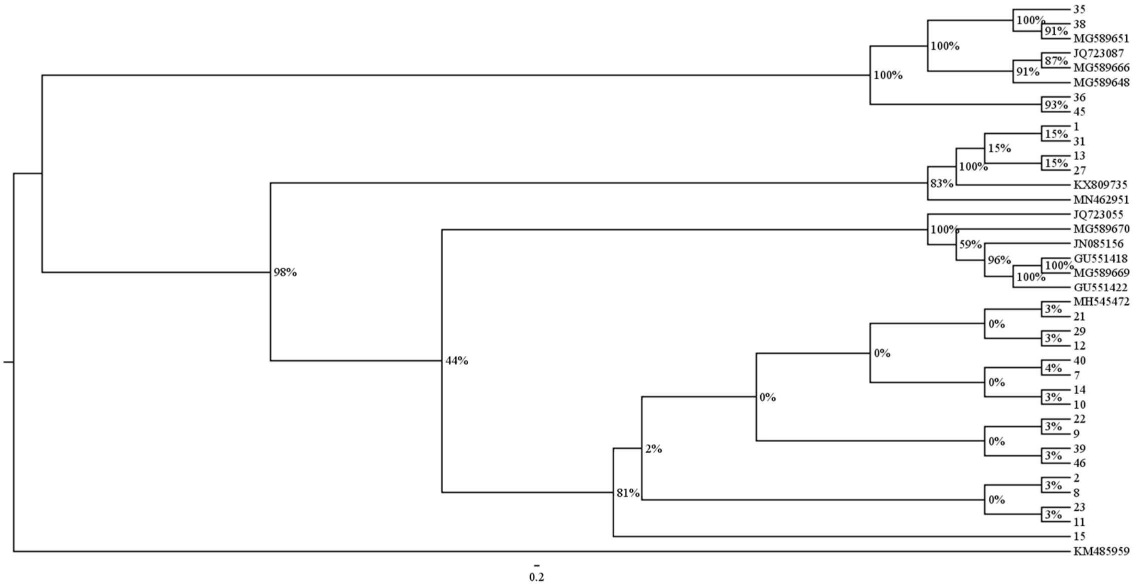

Figure S2. Bayesian phylogenetic tree of the 24 selected Morchella isolates and reference sequences inferred from ITS sequences.

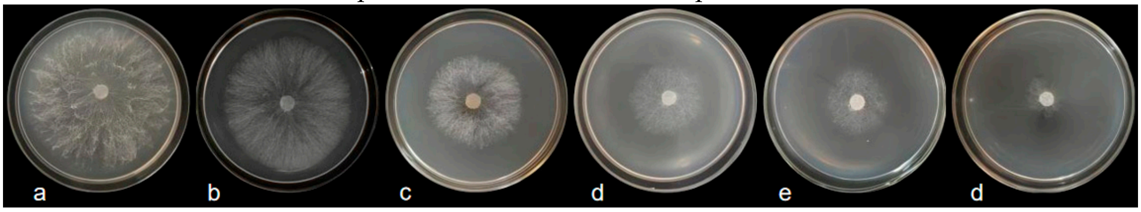

Figure S3. Mycelial growth of *M. sextelata* under different NaCl concentrations. (a) 0 mmol·L^{-1} NaCl; (b) 100 mmol·L^{-1} NaCl; (c) 200 mmol·L^{-1} NaCl; (d) 300 mmol·L^{-1} NaCl; (e) 400 mmol·L^{-1} NaCl; and (f) 500 mmol·L^{-1} NaCl.

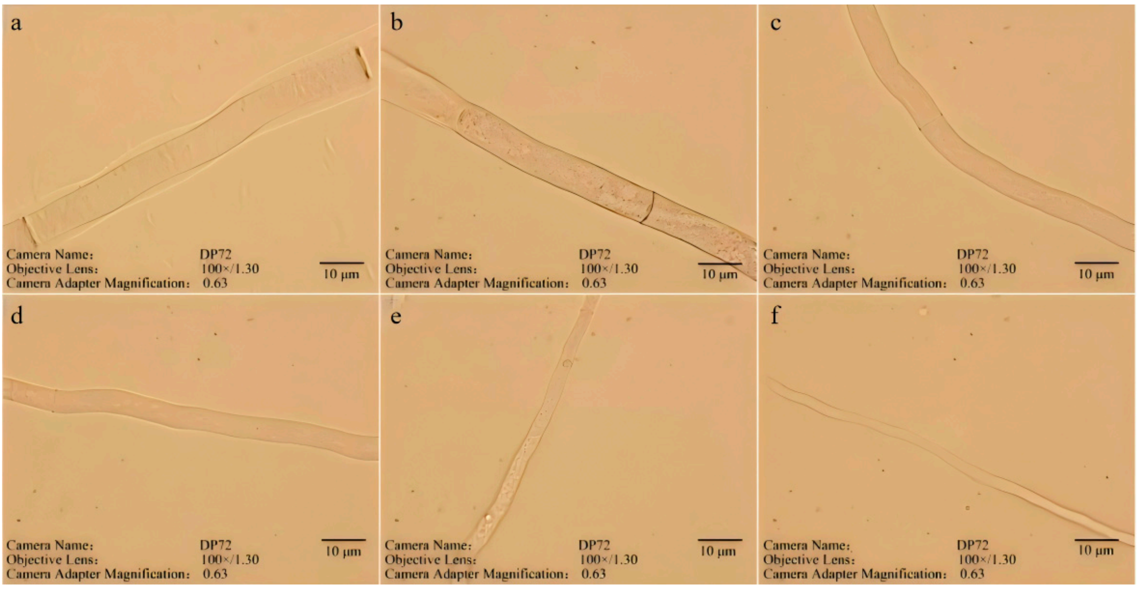

Figure S4. Hyphal morphology of *M. sextelata* under different NaCl concentrations. (a) 0 mmol·L⁻¹ NaCl; (b) 100 mmol·L⁻¹ NaCl; (c) 200 mmol·L⁻¹ NaCl; (d) 300 mmol·L⁻¹ NaCl; (e) 400 mmol·L⁻¹ NaCl; and (f) 500 mmol·L⁻¹ NaCl.

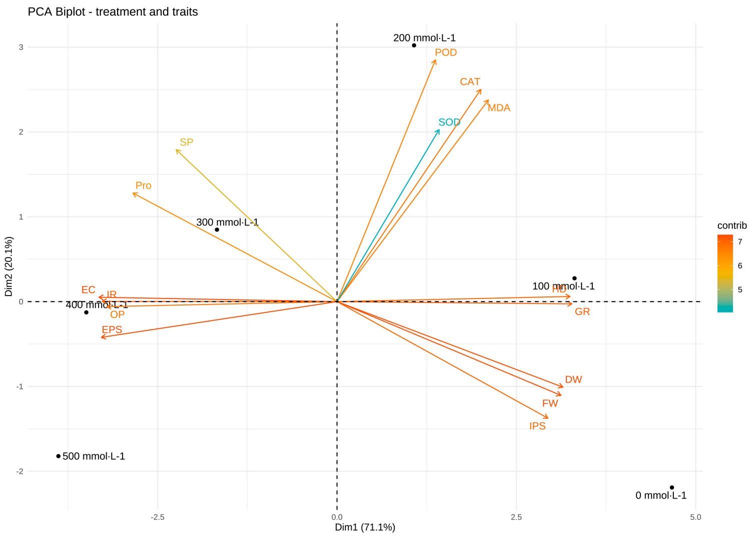

Figure S5. Principal component analysis biplot of NaCl treatments and physiological traits in *M. sextelata* mycelia. The color gradient indicates the relative contribution of each trait to the principal components.

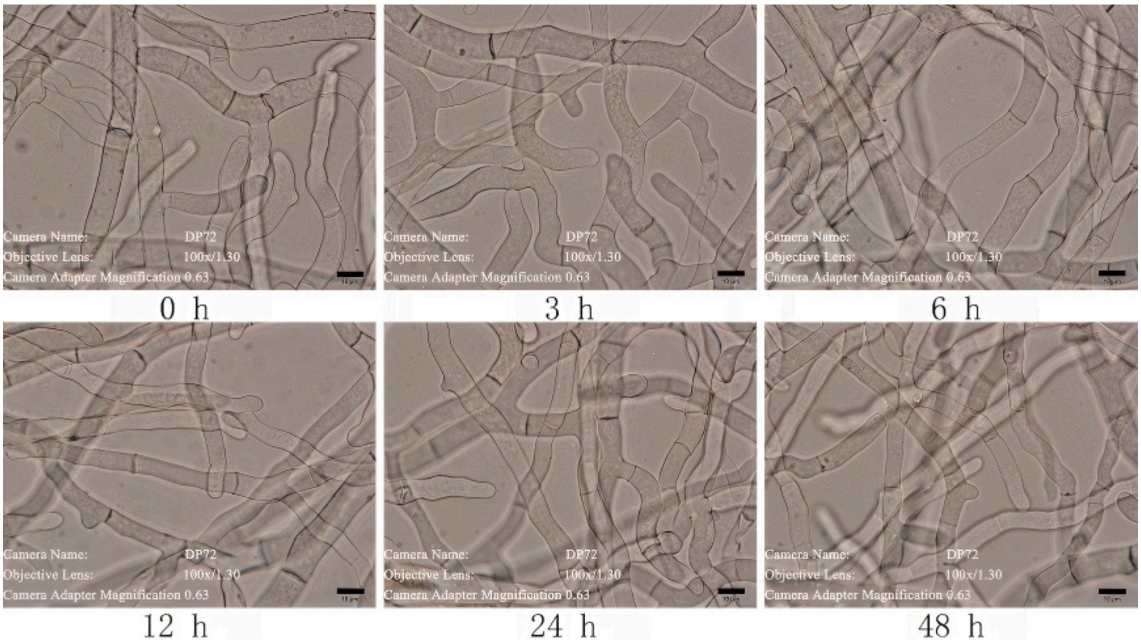

Figure S6. Time-dependent changes in hyphal morphology of *M. sextelata* Mel-21 under 300 mmol·L^{-1} NaCl treatment. Images show mycelial morphology at 0, 3, 6, 12, 24, and 48 h. Scale bar = 10 μm.

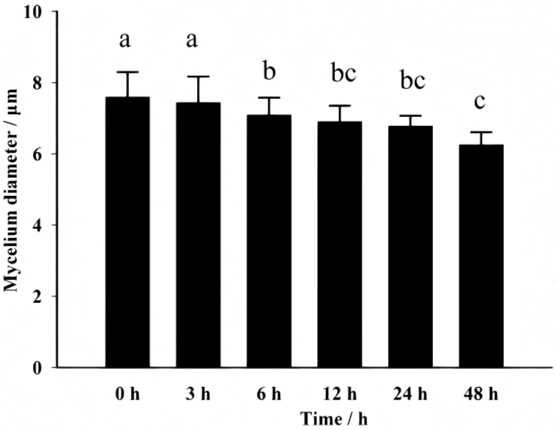

Figure S7. Time-dependent changes in hyphal diameter of *M. sextelata* Mel-21 under 300 mmol·L^{-1} NaCl treatment. Different lowercase letters indicate significant differences among time points at $p < 0.05$.

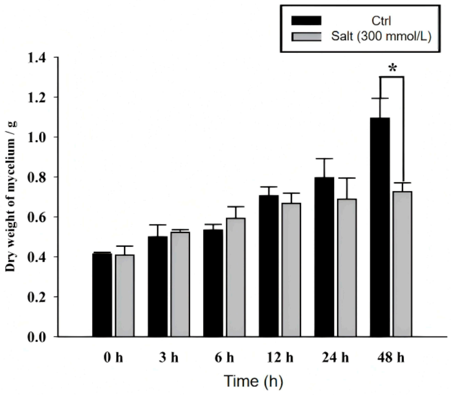

Figure S8. Time-dependent changes in the dry weight of *M. sextelata* Mel-21 mycelia in the control and 300 mmol·L^{-1} NaCl treatment groups

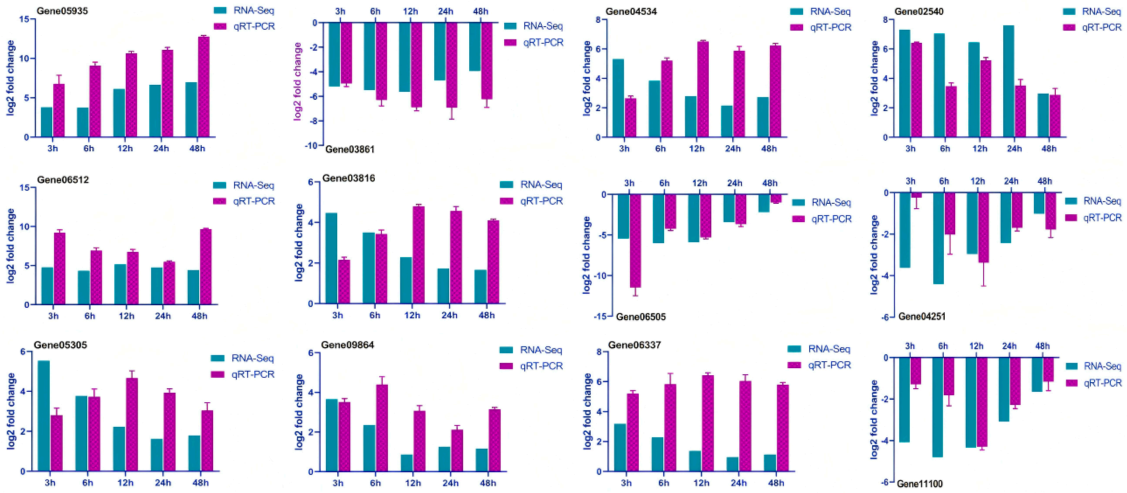

Figure S9. qRT-PCR validation of selected DEGs.

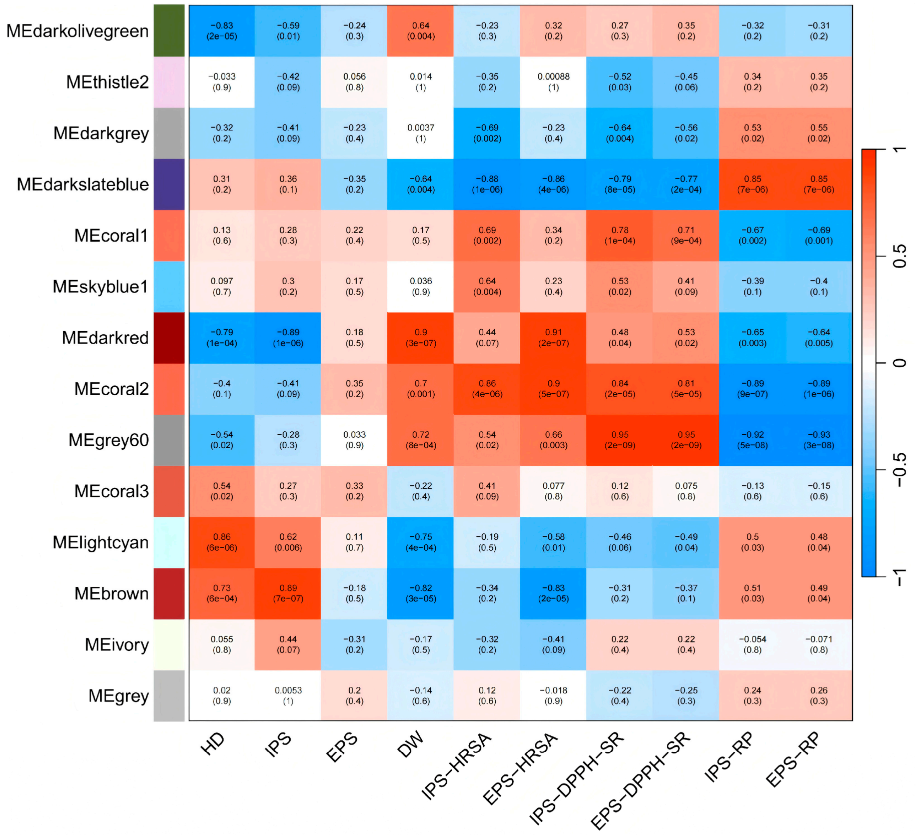

Figure S10. Module–trait relationships identified by weighted gene co-expression network analysis (WGCNA). Each cell shows the Pearson correlation coefficient and its nominal two-sided P value for 18 paired observations. RNA-seq and physiological measurements were obtained from distinct cultures paired at the start of the experiment and matched using the original pairing records. P values were not adjusted for multiple testing, and the associations were interpreted as exploratory. Colors indicate the direction and magnitude of the correlations. HD, hyphal diameter; DW, mycelial dry weight; IPS, intracellular polysaccharide content; EPS, extracellular polysaccharide content; HRSA, hydroxyl radical-scavenging activity; DPPH-SR, DPPH radical-scavenging rate; RP, reducing power.

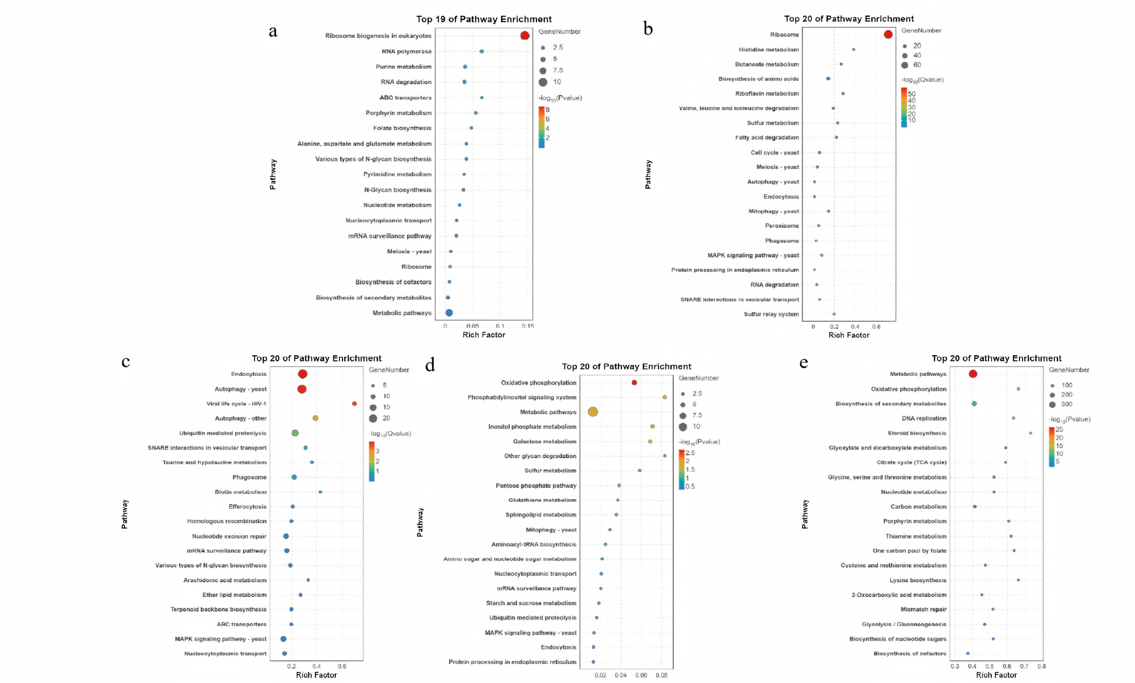

Figure S11 KEGG pathway enrichment analysis of genes in five WGCNA modules selected for their relatively high absolute module–trait correlations. (a) Lightcyan; (b) brown; (c) darkred; (d) grey60; and (e) darkslateblue. Enrichment results are presented descriptively using the nominal $P \leq 0.05$ criterion and do not establish causal relationships between modules and physiological traits.

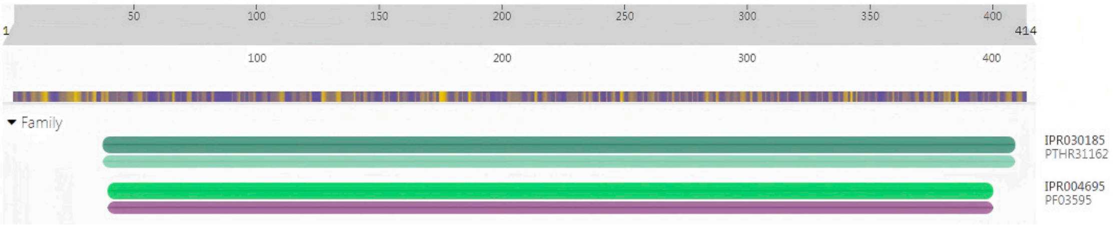

Figure S12. Conserved-domain prediction of the protein encoded by *Gene05935*.

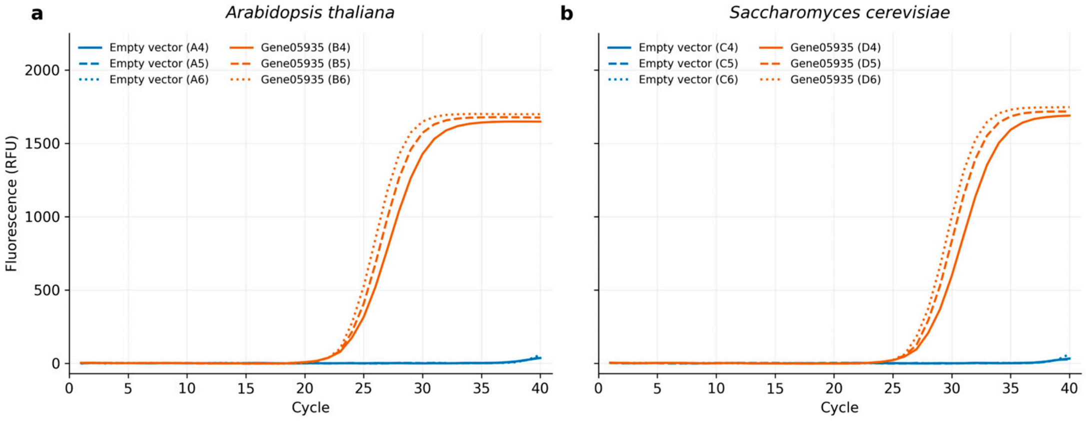

Figure S13. qRT-PCR amplification curves for *Gene05935* in heterologous expression assays. (a) *A. thaliana*; (b) *S. cerevisiae*. Orange curves represent *Gene05935*-expressing samples, and blue curves represent empty-vector controls. .

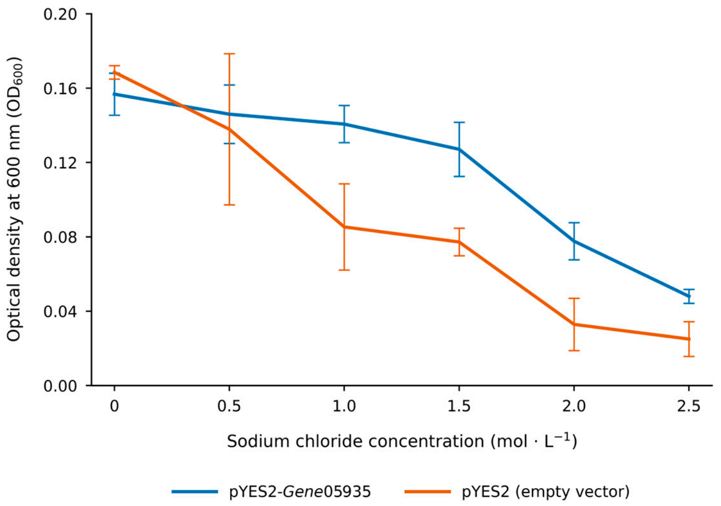

Figure S14 Endpoint growth of Saccharomyces cerevisiae expressing Gene05935 under different NaCl concentrations.

Yeast carrying pYES2-Gene05935 or the empty pYES2 vector was cultured in SC-Ura liquid medium containing 1% galactose and 0–2.5 mol·L^{-1} NaCl at 30 °C for 24 h. Growth was assessed by OD_{600}. Values represent means ± SD from three independent biological replicates (n = 3), with three technical readings averaged for each biological replicate. Lines connect the mean values across NaCl concentrations.

Supplementary Tables

Table S1. Original identifiers and species names recorded at collection for the 48 Morchella isolates used in this study.

Serial number	Strains name	Latin name	Source of strains
1	TL6317	*M. importuna*	Kunming Institute of Botany, Chinese Academy of Sciences
2	SC *sextelata*	*M. sextelata*	Sichuan Academy of Agricultural Sciences
3	*Sextelata* M17	*M. sextelata*	Kunming Institute of Botany, Chinese Academy of Sciences
4	100570	*M. esculenta*	Houshiwu, Tai’an City, Shandong Province
5	*Sextelata* 6-2	*M. sextelata*	Jintang County, Chengdu City, Sichuan Province
6	Y FY4.9	*M. sextelata*	Shandong Yantai Fu’ai Huinong Rare Edible Mushroom Development Co., Ltd.
7	SX *sextelata* A	*M. sextelata*	Yulin City, Shaanxi Province
8	Y 5.30	*M. sextelata*	Kunming Institute of Botany, Chinese Academy of Sciences
9	Y 18-4-9	*M. sextelata*	Sichuan Junyinong Agricultural Sci. and Tech. Co., Ltd.
10	Y 62130	*M. sextelata*	Kunming Institute of Botany, Chinese Academy of Sciences
11	Y M1219	*M. sextelata*	Lijin County, Dongying City, Shandong Province
12	Y 9Y-Z	*M. sextelata*	Lijin County, Dongying City, Shandong Province
13	TL 6-2	*M. importuna*	Yunnan Junshijie Biotechnology Co., Ltd.
14	*Sextelata* 75	*M. sextelata*	Kunming Institute of Botany, Chinese Academy of Sciences
15	Y 20170512LJ	*M. sextelata*	Jintang County, Chengdu City, Sichuan Province
16	Y 20170526	*M. sextelata*	Lijin County, Dongying City, Shandong Province
17	YS	*M. esculenta*	Xiayangjia Village, Guanshui, Muping, Yantai, Shandong, China
18	TL 21-M2	*M. importuna*	Yunnan Junshijie Biotechnology Co., Ltd.
19	Y 20170427	*M. sextelata*	Jintang County, Chengdu City, Sichuan Province
20	YB	*M. importuna*	Kenli District, Dongying City, Shandong Province
21	*Sextelata* 6613	*M. sextelata*	Kunming Institute of Botany, Chinese Academy of Sciences, Yunnan Province
22	20210503	*M. sextelata*	Luoshan Scenic Area, Zhaoyuan City, Yantai, Shandong Province
23	20170326	*M. sextelata*	Weihai Xinbao Food Co., Ltd., Shandong Province
24	120408	*M. esculenta*	Houshiwu, Tai’an City, Shandong Province
25	Y 4-2	*M. sextelata*	Weihai Xinbao Food Co., Ltd., Shandong Province
26	BX	*M. esculenta*	Boxing County, Binzhou City, Shandong Province
27	100536	*M. importuna*	Shandong Bohua High-Efficiency Ecological Agriculture Science and Technology Co., Ltd.
28	120426	*M. esculenta*	Shandong Bohua High-Efficiency Ecological Agriculture Science and Technology Co., Ltd.
29	Y 17-5-22	*M. sextelata*	Weihai Xinbao Food Co., Ltd., Shandong Province
30	TL	*M. importuna*	Sichuan Academy of Agricultural Sciences
31	SC 1	*M. importuna*	Sichuan Academy of Agricultural Sciences
32	SC 2	*M. importuna*	Sichuan Academy of Agricultural Sciences
33	Y 5-5	*M. sextelata*	Weihai Xinbao Food Co., Ltd., Shandong Province

Continued Table S1. Detailed information on tested *Morchella* strains.

Serial number	Strains name	Latin name	Source of strains
34	Y	*M. esculenta*	Guangrao County, Dongying City, Shandong Province
35	20210511	*M. crassipes*	Dadongkuang, Zhifu District, Yantai City, Shandong Province
36	20210425	*M. esculenta*	Daxindian Town, Penglai District, Yantai City, Shandong Province
37	20210427	*M. crassipes*	Yantai Branch, China Agricultural University, Yantai City, Shandong Province
38	661357	*M. crassipes*	Yunnan Junshijie Biotechnology Co., Ltd.
39	6698	*M. sextelata*	Kunming Institute of Botany, Chinese Academy of Sciences
40	62075	*M. sextelata*	Kunming Institute of Botany, Chinese Academy of Sciences
41	2195	*M. importuna*	Jintang County, Chengdu City, Sichuan Province
42	2115	*M. esculenta*	Shangqiu City, Henan Province
43	2633	*M. crassipes*	Laiwu District, Jinan City, Shandong Province
44	2332	*M. crassipes*	Jilin Agricultural University, Jilin Province
45	2196	*M. esculenta*	Mount Tai Scenic Area, Tai'an City, Shandong Province
46	YD-8	*M. sextelata*	Liaocheng City, Shandong Province
47	2194	*M. crassipes*	Tashan Scenic Area, Yantai City, Shandong Province
48	2619	*M. sextelata*	Huangdao District, Qingdao City, Shandong Province

Table S2. Primer sequences used for mating-type gene detection in *Morchella* strains.

Locus	Primer name	Primer sequences(5'→3')	Primer length (nt)
MAT1-1	MAT1-1-1F	AAAACGCTCTATCGCCACCA	20
	MAT1-1-1R	TGCGAAGTTGCGTTTTCAGG	20
MAT1-2	MAT1-2-1F	ATGAAGGCTGTTCGCCAGAA	20
	MAT1-2-1R	TGGTGCTCTCGTGCAGATTT	20

Table S3. Evaluation of germination score under different treatments.

Germination time	Scores
Germination within 24 h	6
Germination within 48 h	5
Germination within 72 h	4
Germination within 96 h	3
Germination within 120 h	2
Germination within 144 h	1

Table S4. Evaluation of mycelial density under different treatments.

Mycelial density	Scores
Dense mycelia, full plate coverage	6
Moderately dense mycelia, full plate coverage	5
Sparse mycelia, full plate coverage	4
Dense mycelia, incomplete plate coverage	3
Moderately dense mycelia, incomplete plate coverage	2
Sparse mycelia, incomplete plate coverage	1

Table S5. Evaluation of sclerotia density under different treatments.

Sclerotia density	Scores
Abundant sclerotia	5
Moderately abundant sclerotia	4
Moderately few sclerotia	3
Few sclerotia	2
No sclerotia	1

Table S6. Primer sequences used for qRT-PCR.

GeneID	Primer sequences（5′→3′）
Gene05935	F：TCCGTGCTGGGCAATATTGT
	R：CAACGCCAAAGACCATTGCA
Gene03861	F：GCATCGACATTGACGCTGTC
	R：CCGTGTAGACCGATCTCGTG
Gene04534	F：CGATGCTGGTTGGGTTTGTG
	R：TTTCAAGAACCGCGTCTCGA
Gene02540	F：GCGCCTTATCGGTATTCCCA
	R：CAGCGCCAATAGACAATGCC
Gene06512	F：GTACGTCGAGCATGCGTCTA
	R：GTGACTCAGTTCGAAGCCCA
Gene03816	F：CAGAAGGCGCAAAGGTAAAG
	R：GGTGACGCTGATTAGGTGGT
Gene06505	F：GATAACTCCATCGCCGAGCA
	R：AAGAACAACCACGCAATGCC
Gene04251	F：TACTACGGGCAAGCAGTGTG
	R：CCTGCCACCCATCAAATCCT
Gene05305	F：ACGGAGTGGTGCTATGGTTC
	R：CTCTGGACACGCTTCTTTCC
Gene09864	F：GGCTGGATTGAGGACTACCA
	R：GATCAAGTGTGGCTCAAGCA
Gene06337	F：CCAGCATCCTCTGCTACTCC
	R：GGGAAAATAGGGGAATGGAA
Gene11100	F：ATCAACTCTCCCTCCTCCCC
	R：GGCCTTTCCACGTCTCTTGA

Table S7. Salt stress response coefficients of physiological indices in 24 *Morchella* strains.

Serial number	GR	MD	SN	FW	DW	FWC	GT
1	0.08	0.17	0.5	0.06	0.06	1.02	0.5
2	0.14	0.17	0.2	0.11	0.12	0.99	0.67
7	0.11	0.5	0.2	0.09	0.11	0.97	0.5
8	0.06	0.33	0.2	0.09	0.1	0.99	0.5
9	0.06	0.17	0.25	0.11	0.1	1.02	0.17
10	0.02	0.17	1	0.1	0.09	1.01	0.17
11	0.09	0.17	0.2	0.11	0.13	0.98	0.5
12	0.07	0.5	1	0.11	0.09	1.03	0.5
13	0.01	0.2	0.5	0.07	0.08	0.98	0.17
14	0.05	0.33	0.2	0.09	0.08	1.02	0.33
15	0.11	0.5	0.5	0.16	0.19	0.98	0.5
21	0.11	1	1	0.37	0.41	0.99	0.5
22	0.09	0.33	0.33	0.09	0.1	0.98	0.5
23	0.13	0.33	0.2	0.1	0.1	1	0.67
27	0.23	0.33	1	0.11	0.12	0.98	0.83
29	0.14	0.17	0.2	0.12	0.14	0.99	0.83
31	0.15	0.17	0.5	0.09	0.1	0.99	0.83
35	0	0	0	0	0	0	0
36	0.02	0.17	0.5	0.06	0.06	1	0.33
38	0.26	0.6	1	0.32	0.43	0.95	0.83
39	0.09	0.17	0.25	0.18	0.17	1.01	0.5
40	0.34	0.75	1	0.11	0.13	0.96	0.83
45	0	0	0	0	0	0	0
46	0.02	0.17	0.5	0.09	0.09	1	0.33
Maximum	0.34	1	1	0.37	0.43	1.03	0.83
Minimum	0	0	0	0	0	0	0
Average	0.1	0.31	0.47	0.11	0.12	0.91	0.48
Standard deviation	0.08	0.24	0.35	0.08	0.1	0.28	0.26
Variable coefficient/%	83.13%	76.52%	73.99%	70.72%	80.90%	30.87%	53.60%

Note: GR is growth rate; MD is mycelial density; SN is sclerotia number; FW is fresh weight; DW is dry weight; FWC is fresh water content; GT is germination time.

Table S8. Correlation matrix of salt tolerance coefficients for individual indices.

Index	GR	MD	SN	FW	FWC	DW	GT
GR	1						
MD	0.58**	1					
SN	0.47*	0.63**	1				
FW	0.45*	0.75*	0.52*	1			
FWC	0.44*	0.39	0.47*	0.47*	1		
DW	0.50*	0.74**	0.52*	0.98**	0.42*	1	
GT	0.86**	0.45*	0.37	0.44*	0.62**	0.46*	1

Note: GR is growth rate; MD is mycelial density; SN is sclerotia number; FW is fresh weight; DW is dry weight; FWC is fresh water content; GT is germination time. "*" and "**" mean significant correlation at 5% and 1% level, respectively.

Table S9. Coefficients and contribution rates of the comprehensive index derived from PCA.

Index	Component1	Component2
GR	0.77	0.46
MD	0.84	-0.26
SN	0.72	-0.12
DW	0.87	-0.39
FW	0.86	-0.41
FWC	0.6	0.33
GT	0.75	0.6
Eigenvalue	4.22	1.08
Contribution rate	60.29	15.44
Cumulative contribution ratio	60.29	75.73

Table S10. Mycelial Na^+ and K^+ contents and K^+/Na^+ mass ratios in *M. sextelata* Mel-21 under different NaCl concentrations with 20 mmol·L⁻¹ KCl supplementation

NaCl concentration（mmol/L）	Na^+ content (mg·g⁻¹ DW)	K^+ content (mg·g⁻¹ DW)	K^+/Na^+
0	0.48 ± 0.04 f	30.51 ± 0.73 c	63.56
100	48.45 ± 1.52 e	50.65 ± 0.55 a	1.05
200	86.30 ± 2.40 d	42.00 ± 0.96 b	0.49
300	101.83 ± 5.60 c	23.83 ± 1.87 d	0.23
400	134.13 ± 3.70 a	19.31 ± 0.90 e	0.14
500	119.67 ± 6.17 b	8.50 ± 0.28 f	0.07

Note: DW, dry weight. All treatments, including the 0 $mmol \cdot L^{-1}$ NaCl control, contained 20 $mmol \cdot L^{-1}$ KCl. Ion contents were determined by ICP-AES. Different lowercase letters within the same ion-content column indicate significant differences among NaCl concentrations ($p < 0.05$; one-way ANOVA followed by Waller–Duncan's multiple-range test). K^+/Na^+ is a dimensionless mass ratio calculated from the mean K^+ and Na^+ contents; no separate variability estimates or statistical comparisons are reported for this ratio.

Table S11. Mycelial Na^+ and Ca^{2+} contents and Ca^{2+}/Na^+ mass ratios in *M. sextelata* Mel-21 under different NaCl concentrations with 20 mmol·L^{-1} $CaCl_2$ supplementation.

NaCl concentration（mmol/L）	Na^+ content ($mg \cdot g^{-1}$ DW)	Ca^{2+} content ($mg \cdot g^{-1}$ DW)	Ca^{2+}/Na^+
0	1.61 ± 0.09 f	19.30 ± 1.02 a	11.99
100	35.00 ± 1.69 e	11.00 ± 0.31 d	0.31
200	86.00 ± 8.71 d	14.50 ± 0.13 b	0.17
300	133.17 ± 6.95 c	12.81 ± 0.08 bc	0.10
400	188.27 ± 6.03 b	12.34 ± 0.18 cd	0.07
500	302.93 ± 19.83 a	11.61 ± 0.53 cd	0.04

Note: DW, dry weight. All treatments, including the 0 mmol·L^{-1} NaCl control, contained 20 mmol·L^{-1} $CaCl_2$. Ion contents were determined by ICP-AES. Different lowercase letters within the same ion-content column indicate significant differences among NaCl concentrations ($p < 0.05$; one-way ANOVA followed by Waller–Duncan's multiple-range test). Ca^{2+}/Na^+ is a dimensionless mass ratio calculated from the mean Ca^{2+} and Na^+ contents; no separate variability estimates or statistical comparisons are reported for this ratio.
